# Genome-Wide Identification and Classification of Soybean C2H2 Zinc Finger Proteins and Their Expression Analysis in Legume-Rhizobium Symbiosis

**DOI:** 10.3389/fmicb.2018.00126

**Published:** 2018-02-06

**Authors:** Songli Yuan, Xiangyong Li, Rong Li, Lei Wang, Chanjuan Zhang, Limiao Chen, Qingnan Hao, Xiaojuan Zhang, Haifeng Chen, Zhihui Shan, Zhonglu Yang, Shuilian Chen, Dezhen Qiu, Danxia Ke, Xinan Zhou

**Affiliations:** ^1^Key Laboratory of Oil Crop Biology, Ministry of Agriculture, Wuhan, China; ^2^Oil Crops Research Institute of Chinese Academy of Agriculture Sciences, Wuhan, China; ^3^Bioinformatics Laboratory, College of Life Sciences, Xinyang Normal University, Xinyang, China

**Keywords:** soybean, C2H2 zinc finger proteins, classification, gene expression, root nodule symbiosis

## Abstract

Root nodule symbiosis (RNS) is one of the most productive and economical systems for nitrogen fixation, and previous studies have shown that several nodule-specific C2H2-zinc finger proteins (ZFPs) play important roles in symbiosis establishment and nodule function. However, C2H2-ZFPs are the most widespread ZFPs in eukaryotes, and a great variation of structure and function exist among the family members. It remains largely unclear whether or not special types of C2H2-ZF genes participate in symbiosis, especially in soybean. In the present study, we performed a genome-wide survey of soybean C2H2-ZF genes, and 321 soybean C2H2-ZF genes were identified and classified into 11 clearly distinguishable subsets (Gm-t1-SF, Gm-t2-SF, Gm-1i-Q-SF, Gm-1i-M-SF, Gm-1i-Z-SF, Gm-1i-D-SF, Gm-2i-Q-SF, Gm-2i-M-SF, Gm-2i-Mix-SF, Gm-3i-SF, and Gm-4i-SF) based on the arrangements, numbers, and types of C2H2-ZF domains. Phylogenetic and gene ontology analyses were carried out to assess the conserved sequence and GO function among these subsets, and the results showed that the classification of soybean C2H2-ZFPs was reasonable. The expression profile of soybean C2H2-ZFPs in multiple tissues showed that nearly half of soybean C2H2-ZFPs within different subsets had expressions in nodules, including a clustering branch consisting of 11 Gm-1i-Q-SF genes specifically expressed in symbiotic-relative tissues. RNA-Seq was used to identify symbiosis-related soybean C2H2-ZFPs, and the expression pattern of the soybean C2H2-ZFPs in roots and nodules at different development stages showed that soybean C2H2-ZFPs mainly played roles in nodule development or nodule function rather than nodulation signal transduction, and nearly half of these genes had high expressions and/or different expression patterns during soybean nodule development, especially for the six clustering branches of genes consisting of different subsets of C2H2-ZFPs. Furthermore, the selected symbiosis-related soybean C2H2-ZFPs might function in legume-rhizobium symbiosis through regulating or interacting with other key proteins. Taken together, our findings provided useful information for the study on classification and conservative function of C2H2-ZFPs, and offered solid evidence for investigation of rhizobium symbiosis-related C2H2-ZFPs in soybean or other legumes.

## Introduction

As one of the most productive and economical systems for nitrogen fixation, root nodule symbiosis (RNS) plays important roles in plant cultivation and fertilizer application (Biswas and Gresshoff, 2014). RNSs are usually found in the N-fixing clade plants, mainly including legume, Cannabaceae (*Parasponia*), and actinorhizal plants (Diedhiou et al., 2014). Legume plants are the third largest family of flowering plants (Cannon et al., 2015) and can form N-fixing symbiosis with rhizobia. The development of legume-rhizobium symbiosis involves changes in the expression of thousands of plant genes (Colebatch et al., 2004; Benedito et al., 2008; Alves-Carvalho et al., 2015; Yuan et al., 2017). Among them, a nodule-specific cysteine-2/histidine-2 (C2H2) zinc finger (ZF) gene (MtRSD) directly participates in normal nodule development and symbiotic nitrogen fixation in *Medicago truncatula* (Sinharoy et al., 2013). Besides, some nodule-specific C2H2-ZF genes play roles in the establishment and functioning of actinorhizal symbioses (Diedhiou et al., 2014). These findings indicate that C2H2-ZF genes play important roles in nodulation and nodule function.

C2H2-zinc finger proteins (ZFPs) are the most widespread ZFPs in higher and lower eukaryotes. In silico analysis has shown that the number of C2H2-ZFPs corresponds to ~2.3, ~3, and 0.8% of all genes in diptera, mammals, and saccharomyces cerevisiae, respectively (Bohm et al., 1997; Chung et al., 2002; Bateman et al., 2004). In plants, the genome-wide analysis has identified 176 C2H2-ZFPs in *Arabidopsis* (Englbrecht et al., 2004), 189 in rice (Agarwal et al., 2007), 124 in foxtail millet (Muthamilarasan et al., 2014), 109 in poplar (Liu et al., 2015), and 118 in tobacco (Minglei et al., 2016). The comparison of the whole C2H2-ZFP sets between plants and other eukaryotes has revealed a remarkable level of complexity through specific diversification and expansion of C2H2-ZF domain. These diversifications and expansions often include four main structural features in C2H2-ZFPs, including the arrangement of C2H2-ZF domains (tandem or dispersed) (Englbrecht et al., 2004), the length of spacer between the ZFs (Klug and Schwabe, 1995; Kubo et al., 1998), the number of C2H2-ZF domains (Englbrecht et al., 2004; Xiong et al., 2015) and the “QALGGH” sequence (Takatsuji, 1999; Liu et al., 2015; Zhang et al., 2016). Moreover, the expansions of C2H2-ZFPs also include the specific non-finger domains (Chung et al., 2002; Urrutia, 2003; Englbrecht et al., 2004). Different classifications or different types of C2H2-ZFPs have been defined in yeast, rice, *Arabidopsis*, petunia, and poplar based on the above-mentioned factors (Bohm et al., 1997; Kubo et al., 1998; Englbrecht et al., 2004; Agarwal et al., 2007; Liu et al., 2015). However, the genome-wide studies and classification of C2H2-ZFPs in legume are quite limited, and whether or not special types of C2H2-ZF genes participate in symbiosis remains largely unexplored, especially in soybean.

In the present study, we classified the 321 identified soybean C2H2-ZFPs and conducted the genome-wide survey of soybean C2H2-ZFPs in legume-rhizobium symbiosis. Expression profiles of different subsets of soybean C2H2-ZFPs in different soybean tissues and symbiosis development stages were used to explore their putative roles in nodule symbiosis. The predicted functional partners and regulatory networks of symbiosis-related soybean C2H2-ZFPs provided useful information or clues for our understanding of the roles in symbiosis of C2H2-ZFPs in soybean or other legumes.

## Materials and methods

### Identification and characteristics of soybean C2H2-ZFPs

The Soybean Genome Database [http://soybase.org/], Phytozome Database [http://www.phytozome.net/soybean], plant transcription factor database (PlantTFDB), and NCBI-BLAST [http://blast.ncbi.nlm.nih.gov/] online resources were searched to identify the entire family of C2H2 ZFPs in Glycine max. All of the identified C2H2 ZFPs subsequently were manually analyzed to confirm the presence of C2H2-ZF domain using SMART database (Letunic et al., 2015). The sequences of 321 soybean C2H2 ZFPs were downloaded from the Phytozome v12.0 (https://phytozome.jgi.doe.gov/pz/portal.html). The molecular weight and isoelectric point (pI) of the deduced polypeptides were calculated using the ExPasy site (http://web.expasy.org/protparam/) (Wilkins et al., 1999). The SoyBase and the Soybean Breeder's Toolbox (https://soybase.org/gb2/gbrowse/gmax2.0/) was searched to identify the synonymous (Ks) of each soybean C2H2-ZFP and duplications of soybean C2H2-ZFPs. The location information of each soybean C2H2-ZF gene on chromosome was determined from the soybean genome annotation file (Gmax_275_Wm82.a2.v1.gene.gff3). Chromosomal location map was constructed using the Circos software (Krzywinski et al., 2009) based on the duplication coordinates defined in the current genome assembly v2.0.

### Subset classification of soybean C2H2-ZFPs

The number, types, and arrangements of ZFs are usually the main factors for defining families and subgroups in C2H2-ZFPs (Bohm et al., 1997; Englbrecht et al., 2004). The identified 321 soybean C2H2-ZFPs were further manually analyzed to search the numbers of C2H2-ZF domain, C2H2-ZF domain sequences and the space length between C2H2-ZFs using SMART database (Letunic et al., 2015). Tandem ZFs were defined as ZFs containing two~nine C2H2-ZF domains, and every two adjacent domains were linked by <12 amino acid residues. ZFs containing a single ZF and/or two~four ZF domains and every two adjacent domains separated by more than 11 amino acid residues were considered as dispersed ZFs. All ZFPs containing two~five tandem ZFs or nine tandem ZFs were assigned accordingly to subsets of Gm-t1-SF or Gm-t2-SF, respectively. Different types of C2H2-ZFs have been defined according to the variation of the plant-specific conserved domain “QALGGH” sequence (Takatsuji, 1999; Liu et al., 2015; Zhang et al., 2016). In this report, C2H2-ZF domains with a plant-specific conserved domain “QALGGH” and a conserved spacing “X2-C-X2-C-X7-QALGGH-X3-H” were classified as Q-type; and those with one~five degraded amino acids in the domain “QALGGH” and certain modifications in the spacing between the two cysteines and two histidines of Q-type were designated as M-type (M1~M5). The Z-type was characterized by the C2H2-ZF domains with more than 12 (Z1) and less than 12 (Z2) in their spacing between the second cysteine and the first histidine. The D-type did not include the second histidine in the C2H2-ZF domain compared with the other three types. According to these defined C2H2-ZF types, C2H2-ZFPs containing a single ZF domain were further classified into four clearly distinguishable subsets (Gm-1i-Q-SF, Gm-1i-M-SF, Gm-1i-Z-SF, and Gm-1i-D-SF). C2H2-ZFPs containing two Q-type or two M-type C2H2-ZF domains were defined as Gm-2i-Q-SF and Gm-2i-M-SF, respectively, while the C2H2-ZFPs containing two different types of C2H2-ZF domains were classified as Gm-2i-Mix-SF. All ZFPs containing three or four dispersed ZFs were classified to subsets of Gm-3i-SF or Gm-4i-SF, respectively.

### Phylogenetic analysis

Multiple sequences alignments of amino acid sequences of soybean C2H2-ZFPs were performed using ClustalW with default parameters in MEGA 6.0 (Tamura et al., 2013). The phylogenetic tree was performed using MEGA6 software with the Neighbor-Joining (NJ) method, and bootstrap analysis was conducted using 1,000 replicates with the p-distance model. For the phylogenetic analysis of the classified soybean C2H2-ZF genes, firstly, classify the subset of each C2H2-ZF gene according to the subset classification of soybean C2H2 ZFPs; secondly, compress the same subset into a subtree; thirdly, flip the subtrees to make aggregation of the same subsets.

### Gene ontology (GO) annotation analysis

The “GO Term Enrichment Tool” in Soybean Genome Database [http://soybase.org/] was used to conducted the GO annotation of soybean C2H2-ZFPs, including biological process, cellular components, and molecular function. The GO slims (subset of GO terms) of soybean C2H2-ZFPs were downloaded from the soybean database (https://soybase.org/goslimgraphic_v2/dashboard.php) and the analysis was performed with a *p*-value of < 0.05.

### Gene expression assay

The expression patterns data of soybean C2H2-ZFPs in various tissues were manually downloaded from plant Phytozome database (Phytozome 12, http://www.phytozome.net/soybean). Our previous RNA-seq data (Yuan et al., 2016a, 2017) was used to analysis the expression patterns of soybean C2H2-ZFPs in rhizobium symbioses. The expression data of *M. truncatula* C2H2-ZFPs in root hairs and nodules were downloaded from *M. truncatula* Gene Expression Atlas database (https://mtgea.noble.org/v3/index.php#__NO_LINK_PROXY__), and the expression data of common soybean C2H2-ZFPs were downloaded from a Common Bean Gene Expression Atlas database (http://plantgrn.noble.org/PvGEA/Download.jsp) Heatmaps of soybean C2H2-ZFPs were produced using the pheatmap packages in R (Han et al., 2016).

### qPCR

The symbiosis-related soybean C2H2s selected from our previous RNA-seq data (Yuan et al., 2016a, 2017) were further evaluated using qPCR, and the methods refer to our previous studies (Yuan et al., 2016b). Briefly, purified RNA samples were treated with DNase I (Takara) and reversely transcribed into cDNA using a Prime Script RT reagent Kit (Perfect Real Time) with gDNA Eraser (Takara Bio, Inc) and oligo (dT) as the primer. Approximately 1 μg of RNA was used for the reverse transcription, then the synthesized cDNA was used as the template for qPCR using primer sets listed in Supplementary Table S7. After an initial denaturation step at for 90°C 30 s, amplifications were carried out with 40 cycles at a melting temperature 90°C of for 5 s, an annealing temperature of 60°C for 15 s, and an extension temperature of 72°C for 12 s, followed by an exrea extension step at 72°C for 5 s. The QACT was selected as the internal control. Sample cycle threshold (CT) values were standardized for each template based on reference gene, and the relative expression of the target gene was calculated using the 2^−ΔΔCT^ method. Each sample was run in triplicate to ensure statistical credibility.

### Protein-protein interaction network analysis

SMART database (Letunic et al., 2015) was searched to identify the predicted functional partners of symbiosis-related soybean C2H2-ZFPs, then classify the genes with similarly interaction network into a group. Each interacting protein was annotated by searching the Soybean Genome Database [http://soybase.org/] and plant Phytozome Database (Phytozome 12, http://www.phytozome.net/soybean).

### Construction of regulatory network of symbiosis-related soybean C2H2-ZFPs

Plant transcriptional regulatory map database (http://plantregmap.cbi.pku.edu.cn/download.php#networks) was searched to identify the regulatory network of the whole soybean C2H2 family members, and then the co-expression network was constructed according to our previous symbiosis-related RNA-seq data (Yuan et al., 2016a) with the Pearson's correlation coefficient *r* > 0.9 and/or *r* < −0.9. The two regulatory networks were intersected to obtain the primary expression regulatory network. The nodule development-related RNA-seq data of our previous (Yuan et al., 2017) were used to remove the soybean C2H2-ZFPs that are not related to nodule development and/or nodule function, then the redundant genes were removed, and the ultimate regulatory network was obtained. The regulatory network was constructed using Cytoscape software (http://www.cytoscape.org/).

## Results

### Identification of C2H2-ZFPs in soybean

**Figure 1:**
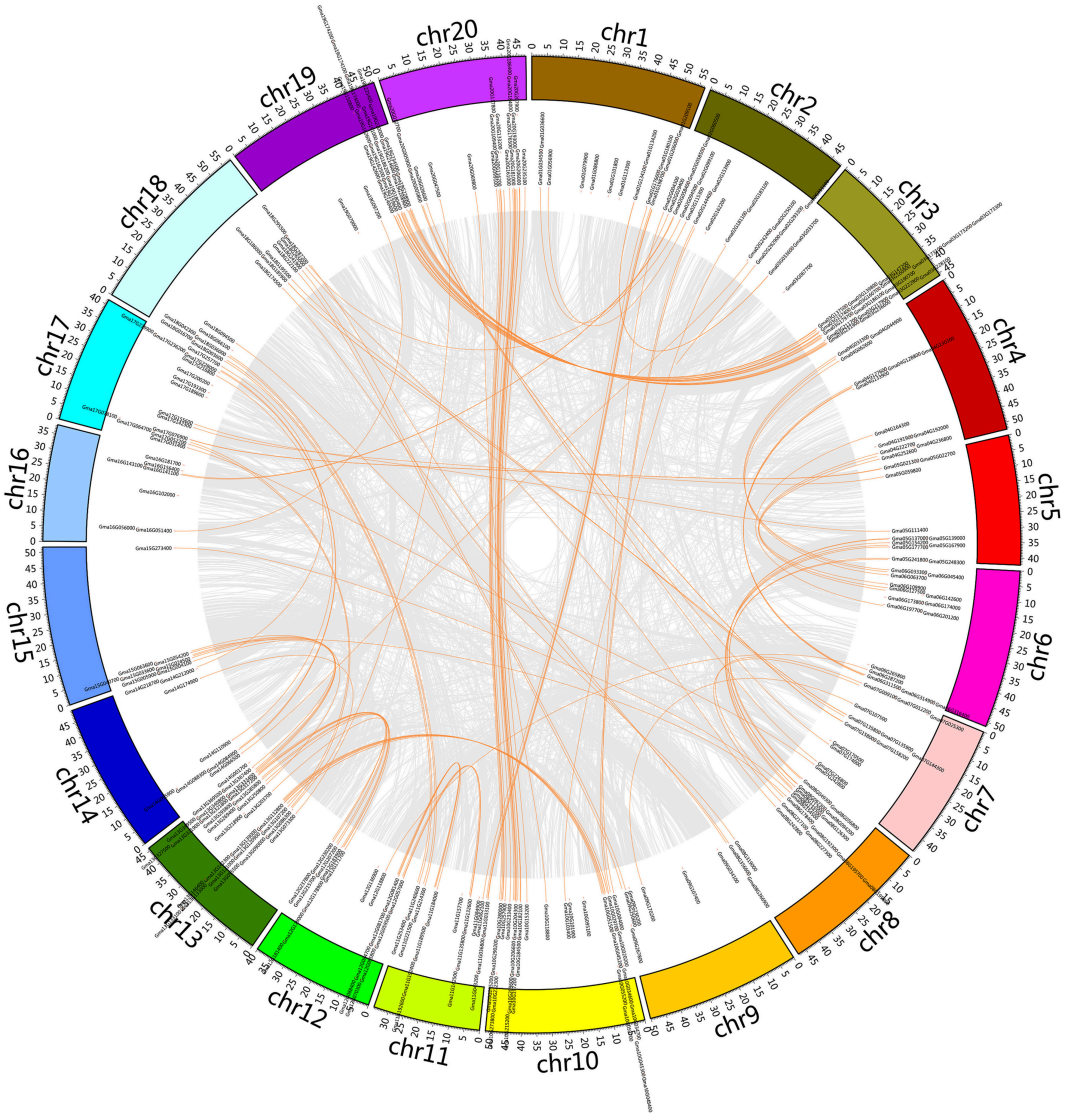
Chromosomal location of the soybean C2H2-ZF genes. The illustrated genome-wide chromosome organization caused by whole genome duplication events is accomplished using the Circos software based on the duplication coordinates defined in the soybean genome assembly v2.0. Segmental duplicated blocks are gray coded. Paralogous pairs of soybean C2H2-ZF genes are connected with orange lines.

Soybean genome database and plant transcription factor database (PlantTFDB) were searched, and 345 candidate C2H2-ZFPs were preliminary identified in the Glycine max var. Williams 82 genome. All of these candidates were manually analyzed using the SMART database to verify the presence of the C2H2-ZF domain, and 321 C2H2-ZF genes were finally identified (Figure 1 and Supplementary Table S1). This number was more than that present in PlantTFDB, where 239 C2H2-ZFPs have been deposited for soybean, and Supplementary Table S2 shows the ID information of those 82 C2H2-ZF genes that were not present in PlantTFDB. The molecular masses of identified soybean C2H2-ZFPs varied from 9,732.2 to 179,775.96 Da. The encoded proteins varied from 87 to 1,592 amino acid residues, and their corresponding isoelectric points (pI) ranged from 4.98 to 9.88 (Supplementary Table S1). Moreover, these 321 soybean C2H2-ZFPs were unevenly distributed on the 20 soybean chromosomes and ChrU, and chromosome 10 had the largest number (30) of C2H2-ZF genes, followed by 28 on chromosome 13 (Figure 1 and Supplementary Table S1).

To determine the possible relationship between the soybean C2H2-ZF genes and potential duplications, all of the whole-genome duplication (WGD)-derived paralogs (including soybean C2H2-ZF genes) were plotted against chromosome position (Figure 1), and Supplementary Table S1 shows the detailed information of soybean C2H2-ZF genes. About 7.79% (25 of 321) of soybean C2H2-ZFPs were not involved in the glycine recent duplication event. In contrast, the other 296 genes were preferentially retained duplicates. Moreover, 135 pairs (274 genes, paralogs, and tandem duplications) were retained both duplicates, and 22 single genes were retained only one duplicate, whereas the other became a pseudogene C2H2-ZFP or was lost. Most paralogs of soybean C2H2-ZF genes were localized in the large segment replication regions (Figure 1), suggesting that these duplication events were mainly caused by duplication of chromosomal large segment. Besides, the soybean genome database [http://soybase.org/] was searched to identify the Ks values of these C2H2-ZFPs created by the glycine recent duplication event (Supplementary Table S1). Most of the paralogs localized on the same two chromosomes had the same Ks value, indicating that these duplication events occurred at the same time.

### General subset classification of C2H2-ZFPs in soybean

To investigate the characteristics of the C2H2-ZF domains in soybean, we manually identified all of the C2H2-ZF domains of 321 soybean C2H2-ZFPs using the SMART database, and most of the C2H2-ZF domains could be represented as X2-Cys-X(1~4)-Cys-X12-His-X(1~8)-His, where X represents any amino acid and the number indicates the consensus spacing between the conserved amino acid residues (Supplementary Tables S3, S4). Different types of C2H2-ZF domains were defined (described in materials and methods), and the details were shown in Supplementary Table S3.

**Figure 2:**
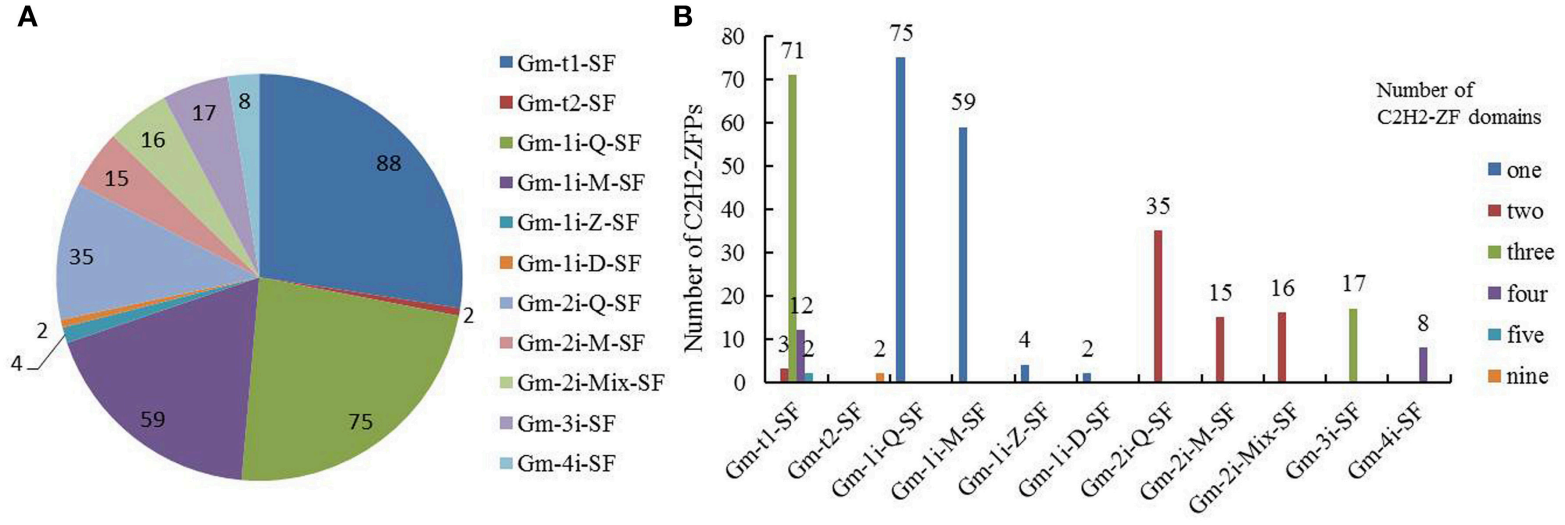
Subset classification of C2H2-ZF genes in the soybean genome. **(A)** Numbers of C2H2-ZFPs in the 11 different subsets. **(B)** Numbers of C2H2-ZFPs containing 1~5 and 9 C2H2-ZF domains. For the detailed information of the classified C2H2-ZFPs in soybean, see Supplementary Tables S3, S4.

In soybean, 90 C2H2-ZFPs contained tandem C2H2-ZF arrays with 88 proteins in Gm-t1-SF and two proteins in Gm-t2-SF, and the 231 proteins (about 72%) with isolated C2H2-ZF domains were further classified into nine clearly distinguishable subsets (Gm-1i-Q-SF, Gm-1i-M-SF, Gm-1i-Z-SF, Gm-1i-D-SF, Gm-2i-Q-SF, Gm-2i-M-SF, Gm-2i-Mix-SF, Gm-3i-SF, Gm-4i-SF) according to the numbers and types of C2H2-ZF domains (Figure 2 and Supplementary Table S4). Most of the C2H2-ZFPs in Gm-t1-SF had three C2H2-ZF domains (about 80.7%), 12 genes had four C2H2-ZF domains, and only two or three genes possessed two or five C2H2-ZF domains (Figure 2B). Most of Gmisolated-SF proteins (140) contained a single C2H2-ZF domain, followed by 66 proteins containing two C2H2-ZF domains, 17 proteins containing three C2H2-ZF domains and eight proteins containing four C2H2-ZF domains (Figure 2B). Besides, the Gmtandem-SF proteins had no Q-type C2H2-ZF domain in their C2H2-ZF domains (Supplementary Table S4), which is similar with the C2H2-ZFPs of yeast and animal (Takatsuji, 1999). Among the Gmisolated-SF proteins, more than half of C2H2-ZFPs contained one or more Q-type C2H2-ZF domains (Supplementary Table S4). In addition, 86 proteins contained one Q-type C2H2-ZF domain (75 in Gm-1i-Q-SF, two in Gm-2i-Mix-SF and nine in Gm-3i-SF), followed by 43 proteins containing two Q-type C2H2-ZF domains (35 in Gm-2i-Q-SF, four in Gm-3i-SF and Gm-4i-SF, respectively) and one protein containing three Q-type C2H2-ZF domains (Gm-3i-SF).

### Phylogenetic analysis of the classified soybean C2H2-ZF genes

**Figure 3:**
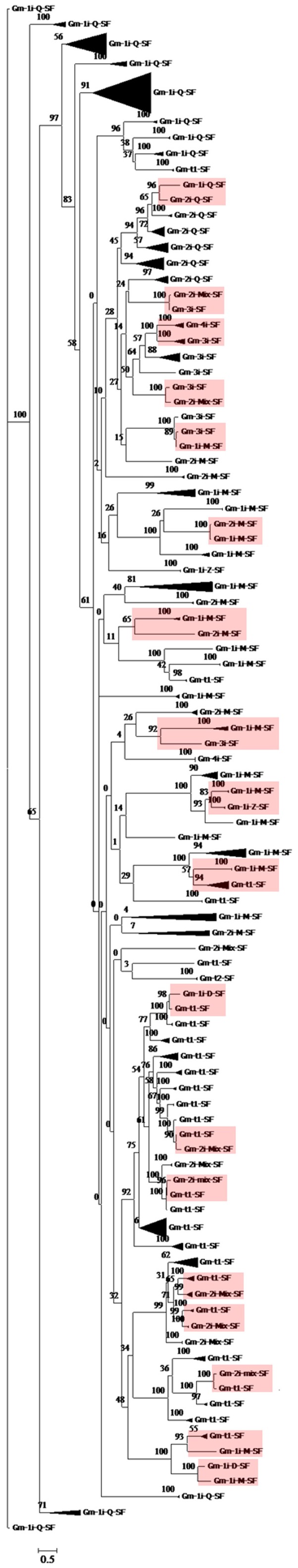
Phylogenetic analysis of the classified soybean C2H2-ZF genes. Firstly, neighbor-joining phylogenetic tree of the 321 soybean C2H2-ZFPs was constructed using MEGA6 by the neighbor-joining method with 1,000 bootstrap replicates; then classified the subset of each C2H2-ZF gene and compressed the subtree of the neighbor-joining phylogenetic tree to construct a phylogenetic tree of the 11 classified soybean C2H2-ZFP subsets. The low bootstrap supports of some main branches indicated that a high degree of complexity was existed in different soybean C2H2-ZFPs subsets. Most of the same subset C2H2-ZFPs was clustered together and had more than 50% bootstrap support. RGB background indicated 18 small branches with different subsets had significant bootstrap support (>50%).

In the present study, we investigated the phylogenetic relationships of the above-mentioned 11 classified C2H2-ZF gene families. First, we constructed a neighbor-joining phylogenetic tree using the full-length amino acid sequences of the 321 soybean C2H2-ZFPs. Second, we classified the subset of each C2H2-ZF gene and compressed the subtree using the MEGA version 6.0 program. The results were shown in Figure 3, and the detailed gene ID information was shown in Supplementary Figure S1. Based on the phylogenetic analysis, the bootstrap supports of some main branches were very low, while most of C2H2-ZFPs in the same subset were clustered together and had more than 50% bootstrap support (Figure 3 and Supplementary Figure S1). These results indicated that a great variation of structure existed among different subsets of soybean C2H2-ZFPs, the sequences were relatively conservative among different members of C2H2-ZFPs in the same subset, and the above-mentioned classification was reasonable.

Besides, 18 small branches with different subsets also had significant bootstrap supports (>50%), including 10 different groups (Gm-1i-Q-SF and Gm-2i-Q-SF; Gm-2i-Mix-SF and Gm-3i-SF; Gm-1i-M-SF and Gm-2i-M-SF; Gm-1i-M-SF and Gm-3i-SF; Gm-1i-M-SF and Gm-1i-Z-SF; Gm-1i-D-SF and Gm-t1-SF; Gm-1i-M-SF and Gm-1i-D-SF; Gm-1i-M-SF and Gm-t1-SF; Gm-2i-Mix-SF and Gm-t1-SF; Gm-3i-SF and Gm-4i-SF; Figure 3 and Supplementary Figure S1). Among them, eight groups consisted of the C2H2-ZFPs with different numbers of C2H2-ZF domains, three groups (Gm-1i-D-SF and Gm-t1-SF; Gm-1i-M-SF and Gm-t1-SF; Gm-2i-Mix-SF and Gm-t1-SF) were composed of the C2H2-ZFPs with tandem and/or dispersed C2H2-ZF domains, and two groups (Gm-1i-M-SF and Gm-1i-D-SF; Gm-1i-M-SF and Gm-1i-Z-SF) contained different types of C2H2-ZF domains (except for Q-type). Interestingly, one group was composed of the C2H2-ZFPs with only one or two Q-type C2H2-ZF domains (Figure 3). These results suggested that the C2H2-ZFPs with only one or two Q-type C2H2-ZF domains were relatively conservative in soybean.

### Go analysis of soybean C2H2-ZFPs

**Figure 4:**
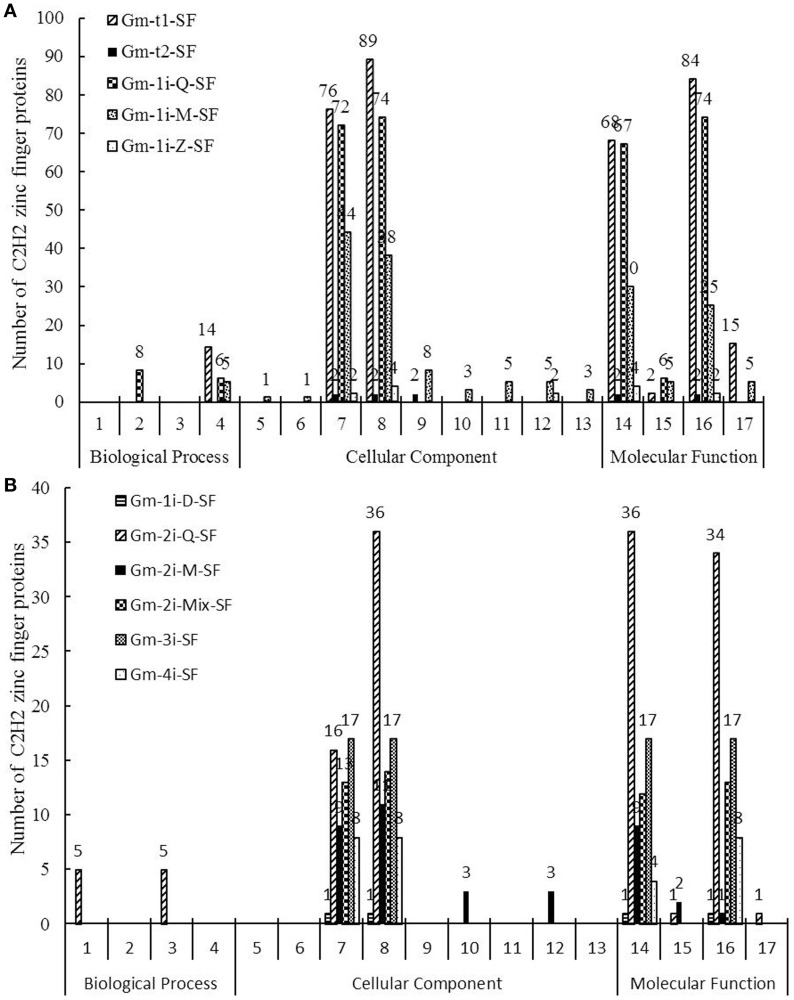
Gene Ontology (GO) results for the 11 soybean C2H2-ZFP subsets. Go analysis of 321 soybean C2H2-ZFPs (11 subsets) showed their involvement in biological process, cellular components, and molecular function, and the numbers of genes in each term are shown in histograms. Seventeen GO function terms are indicated: 1, Signal Transduction; 2, Flower Development; 3, Photosynthesis; 4, Cell Differentiation; 5, Extracellular Region; 6, Cell Wall; 7, Intracellular; 8, Nucleus; 9, Nucleolus; 10, Cytoplasm; 11, Mitochondrion; 12, Cytosol; 13, Plasma Membrane; 14, Nucleic Acid Binding; 15, DNA Binding; 16, Sequence-specific DNA Binding Transcription Factor Activity; 17, Protein Binding. **(A)** Gm-t1-SF, Gm-t2-SF, Gm-1i-Q-SF, Gm-1i-M-SF and Gm-1i-Z-SF. **(B)** Gm-1i-D-SF, Gm-2i-Q-SF, Gm-2i-M-SF, Gm-2i-Mix-SF, Gm-3i-SF, and Gm-4i-SF.

GO was used to classify the C2H2-ZFPs in above-mentioned 11 subsets. A total of 17 GO function terms were listed in Figure 4 and divided into three categories as follows: biological process, cellular component and molecular function. Supplementary Table S5 lists the detailed gene ID information of these 17 GO function terms. The biological process was associated with only four soybean C2H2-ZFP subsets, which mainly focused on cell differentiation, flower development, signal transduction, and photosynthesis. For all 11 subsets, prediction of cellular components showed that most of the genes were localized in intracellular compartments (260 genes) and nucleus (294 genes). Besides, Gm-1i-Z-SF genes were also localized in cytosol, Gm-2i-M-SF genes were localized in cytosol and cytoplasm, Gm-t2-SF genes were localized in nucleolus, and most notably, Gm-1i-M-SF genes were localized in multiple cellular components, including cytosol, nucleolus, cytoplasm, mitochondrion, plasma membrane, extracellular region, and cell wall. The main molecular functions of most of the C2H2-ZFPs in the 11 subsets were sequence-specific DNA binding transcription factor activity (261 genes) and nucleic acid binding (250 genes). Beyond these two molecular functions, five subsets (Gm-t1-SF, Gm-1i-Q-SF, Gm-1i-M-SF, Gm-2i-Q-SF, and Gm-2i-M-SF) could also participate in DNA binding, and three subsets (Gm-t1-SF, Gm-1i-M-SF, and Gm-2i-Mix-SF) played roles in protein binding.

C2H2-ZFPs differ widely in their functions, and they are involved in multiple physiological processes and stress responses in plants (Takatsuji, 1999; Huang et al., 2004; Ciftci-Yilmaz and Mittler, 2008; Liu et al., 2015). To predict the biological function of soybean C2H2-ZFPs, we performed Go enrichment analysis of G. max genome in soybean genome database [http://soybase.org/]. A total of 32 Go slim terms (subset of GO terms) significantly (*P* < 0.05) over/under-represented in these genes compared with all the annotated genes of genome (Table 1), and the detailed gene ID information was shown in Supplementary Table S6. These Go slim terms were related to a wide range of processes, including regulation of transcription, regulation of post-translational modification, signal transduction, cell differentiation, development of tissues and organs, and response to various stimuli. Besides, more than 65.6% Go slim terms had only one subset of C2H2-ZFPs, indicating that the biological functions were relatively conservative among the classified 11 subsets of C2H2-ZFPs.

**Table 1:** GO slim terms in soybean C2H2-ZFPs compared with all the annotated genes of genome.

**GO term ID**	**Subsets and numbers**	**Over/Under-represented**	**Corrected *P*-value**	**GO Description**
GO:0006355	218	Overrepresented	1.30929E-42	Regulation of transcription, DNA-dependent
GO:0051973	3 (Gm-1i-Q-SF)	Overrepresented	0.021678694	Positive regulation of telomerase activity
GO:0006417	12 (Gm-1i-Q-SF, Gm-t1-SF and Gm-1i-M-SF)	Overrepresented	1.20782E-06	Regulation of translation
GO:1900109	4 (Gm-t1-SF)	Overrepresented	2.1153E-05	Regulation of histone H3-K9 dimethylation
GO:0035067	4 (Gm-t1-SF)	Overrepresented	0.000307881	Negative regulation of histone acetylation
GO:0033169	6 (Gm-t1-SF)	Overrepresented	5.08703E-08	Histone H3-K9 demethylation
GO:0006457	2 (Gm-2i-M-SF)	Underrepresented	0.015078646	Protein folding
GO:0007165	5 (Gm-2i-Q-SF)	Underrepresented	0.000326855	Signal transduction
GO:0009965	27 (Gm-1i-Q-SF, Gm-t1-SF and Gm-1i-M-SF)	Overrepresented	0.000149664	Leaf morphogenesis
GO:0030154	25 (Gm-1i-Q-SF, Gm-t1-SF and Gm-1i-M-SF)	Overrepresented	6.47367E-07	Cell differentiation
GO:2000011	3 (Gm-1i-M-SF)	Overrepresented	0.001130818	Regulation of adaxial/abaxial pattern formation
GO:0010500	10 (Gm-2i-Mix-SF and Gm-t1-SF)	Overrepresented	1.54005E-11	Transmitting tissue development
GO:0010026	8 (Gm-1i-Q-SF)	Overrepresented	0.000356462	Trichome differentiation
GO:0010093	8 (Gm-1i-Q-SF)	Overrepresented	0.01569553	Specification of floral organ identity
GO:2000904	6 (Gm-t1-SF)	Overrepresented	1.13217E-05	Regulation of starch metabolic process
GO:0010160	6 (Gm-1i-Q-SF)	Overrepresented	1.94968E-05	Formation of organ boundary
GO:0035264	5 (Gm-2i-Q-SF)	Overrepresented	3.95396E-07	Multicellular organism growth
GO:0009793	4 (Gm-1i-Q-SF and Gm-1i-Z-SF)	Underrepresented	0.000676858	Embryo development ending in seed dormancy
GO:0045604	4 (Gm-t1-SF)	Overrepresented	0.000307881	Regulation of epidermal cell differentiation
GO:0010117	5 (Gm-2i-Q-SF)	Overrepresented	0.000448948	Photoprotection
GO:0010031	5 (Gm-t1-SF)	Overrepresented	0.002027841	Circumnutation
GO:0009959	5 (Gm-t1-SF)	Overrepresented	0.01248965	Negative gravitropism
GO:0009611	5 (Gm-2i-Q-SF)	Underrepresented	0.031241483	Response to wounding
GO:0009733	5 (Gm-2i-Q-SF)	Underrepresented	0.043936403	Response to auxin stimulus
GO:0002679	21 (Gm-2i-Q-SF, Gm-1i-Q-SF and Gm-1i-M-SF)	Overrepresented	0.009990945	Respiratory burst involved in defense response
GO:0048579	4 (Gm-1i-Q-SF and Gm-t1-SF)	Overrepresented	0.002472123	Negative regulation of long-day photoperiodism, flowering
GO:0010106	17 (Gm-t1-SF,Gm-2i-Mix-SF and Gm-1i-Q-SF)	Overrepresented	0.0005255	Cellular response to iron ion starvation
GO:0006826	17 (Gm-t1-SF,Gm-2i-Mix-SF and Gm-1i-Q-SF)	Overrepresented	0.000657982	Iron ion transport
GO:0009651	9 (Gm-1i-M-SF and Gm-2i-Q-SF)	Underrepresented	5.40743E-05	response to salt stress
GO:0009937	6 (Gm-t1-SF)	Overrepresented	2.00227E-07	Regulation of gibberellic acid mediated signaling pathway
GO:0010447	5 (Gm-t1-SF)	Overrepresented	3.95396E-07	Response to acidity
GO:0009590	5 (Gm-t1-SF)	Overrepresented	0.00028063	Detection of gravity

### Expression profile of soybean C2H2-ZFPs in multiple tissues and symbiosis with *B. japonicum* strain 113-2

**Figure 5:**
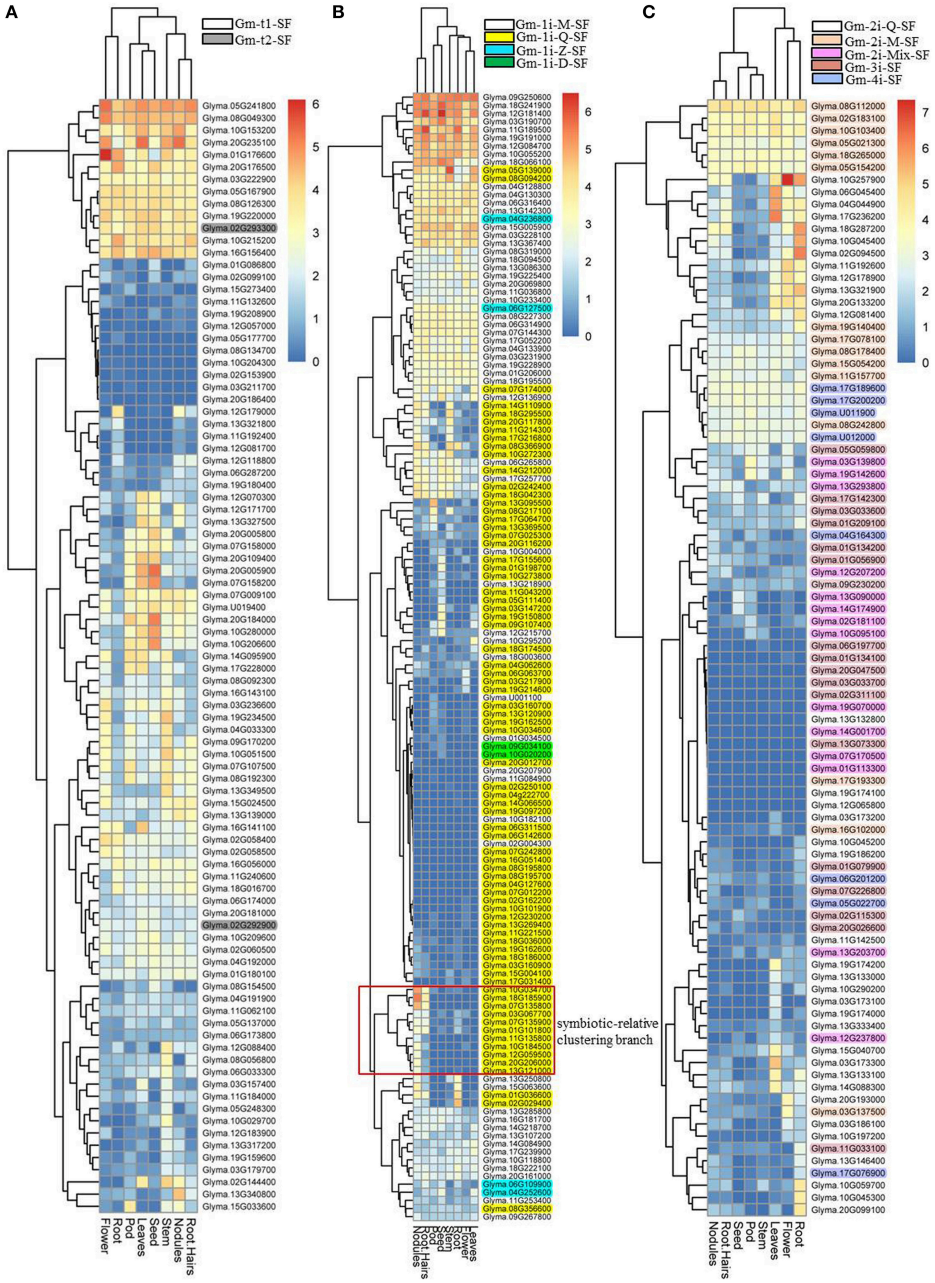
Expression analysis of 321 soybean C2H2-ZFPs in different tissues in the Phytozome database. Heatmaps were produced based on the expression values of soybean C2H2-ZFPs in the Phytozome database using the pheatmap packages in R. These tissues include flower, leaves, nodules, pod, root, root hairs, seed, and stem. The red frame indicates the symbiotic-relative clustering branch. **(A)** Heatmap showing Gm-t1-SF and Gm-t2-SF genes in eight selected tissues. **(B)** Heatmap showing Gm-1i-Q-SF, Gm-1i-M-SF, Gm-1i-Z-SF, and Gm-1i-D-SF genes in eight selected tissues. **(C)** Heatmap showing Gm-2i-Q-SF, Gm-2i-M-SF, Gm-2i-Mix-SF, Gm-3i-SF, and Gm-4i-SF in eight selected tissues.

Based on plant phytozome database (http://www.phytozome.net/soybean), we investigated the expression profiles of the 11 classified soybean C2H2-ZF gene families in various tissues, including flower, leaf, nodules, pod, root, root hair, seed, and stem. Figure 5 showed that the soybean C2H2-ZF gene families had different expression patterns in multiple tissues. Two Gm-t2-SF genes and most of Gm-t1-SF genes, Gm-1i-M-SF genes, and Gm-2i-M-SF genes were highly expressed in all or most of tested tissues (Figure 5). Two Gm-1i-D-SF genes, half of Gm-1i-Z-SF genes and Gm-4i- SF genes, and most of the Gm-3i- SF genes and Gm-2i-Mix-SF genes had no or extremely low expression in these tissues (Figures 5B,C). For Gm-1i-Q-SF genes, most of them had no or extremely low expression in all of the tested tissues (Figure 5B). Most of the Gm-2i-Q-SF genes were specifically expressed in one or more tissues (Figure 5C), indicating that the expression pattern or biological function of these C2H2-ZF genes was tissue specific. Besides, it is worth noting that nearly half of these C2H2-ZFPs within different subsets were expressed in nodules, including a clustering branch consisting of 11 Gm-1i-Q-SF genes specifically expressed in symbiotic-relative tissues (Figure 5B). These findings suggested that soybean C2H2-ZFPs played important roles in rhizobial symbiont.

**Figure 6:**
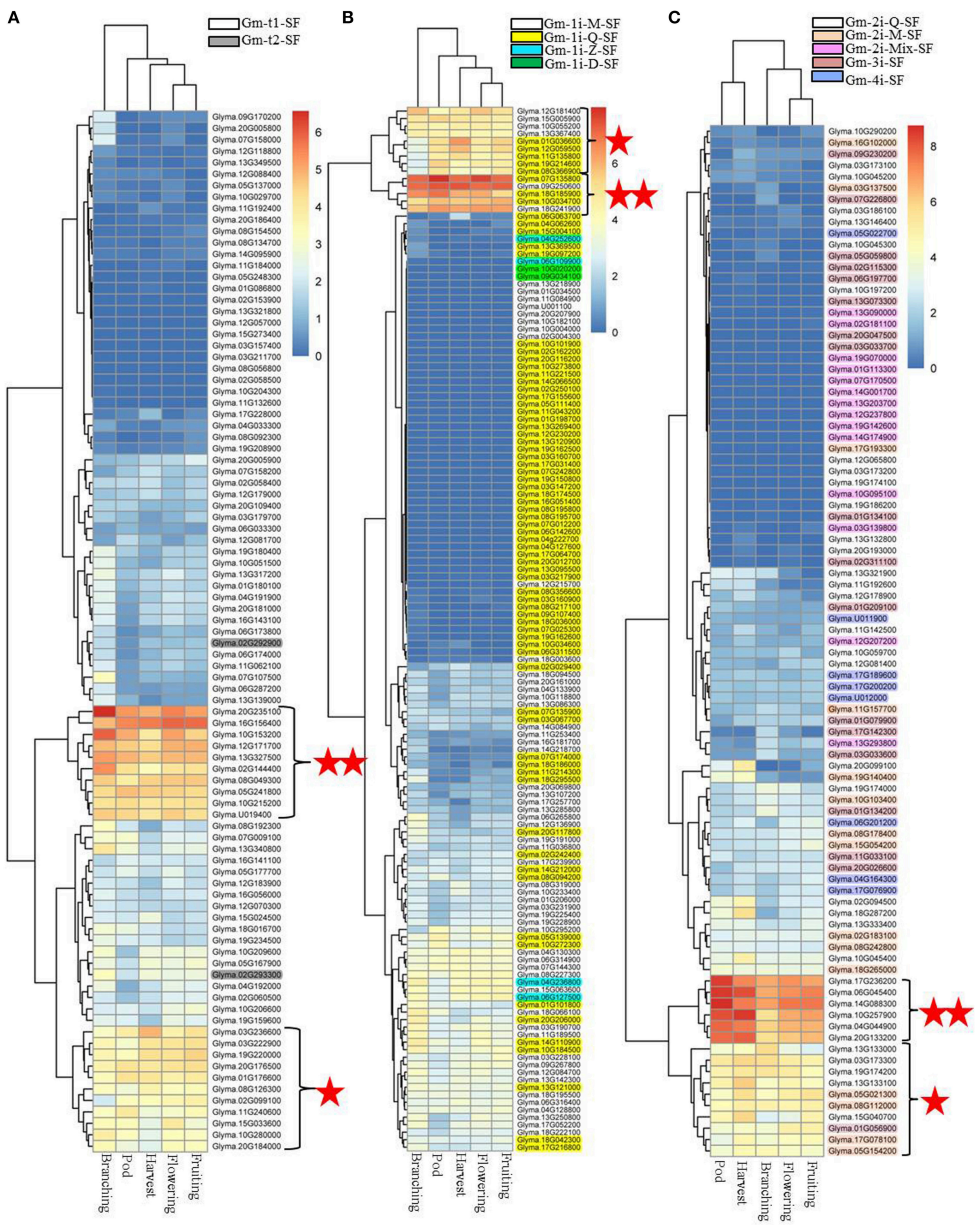
RNA-seq analysis of 321 soybean C2H2-ZFPs in soybean nodules at five important developmental stages. Heatmaps were produced based on our RNA-Seq results using the pheatmap packages in R. The description of these five important developmental stages (branching stage, flowering stage, fruiting stage, pod stage, and harvest stage) were shown in our previous research (Yuan et al., 2017). “⋆⋆”indicates the clustering branches of genes were expressed most abundantly in all or most of above-mentioned nodule samples. “⋆”indicates the clustering branches of genes were expressed high in nodule samples. **(A)** Heatmap for Gm-t1-SF and Gm-t2-SF genes. **(B)** Heatmap for Gm-1i-Q-SF, Gm-1i-M-SF, Gm-1i-Z-SF and Gm-1i-D-SF genes. **(C)** Heatmap for Gm-2i-Q-SF, Gm-2i-M-SF, Gm-2i-Mix-SF, Gm-3i-SF, and Gm-4i-SF genes.

To determine which types of soybean C2H2-ZFPs played roles in nodulation and/or nodule development, we analyzed the expression profiles of the 11 classified subsets of C2H2-ZFPs in roots and nodules at different developmental stages by RNA-Seq, and the results were shown in Supplementary Figure S2 and Figure 6. We compared the expression levels of soybean C2H2-ZF genes in roots (CK and inoculated with *B. japonicum* strain 113-2) at 0.5 h, 7–24 h, 5 days, 16 days, and 21 days post-inoculation. The results showed that only small part of genes were induced by inoculation at some time points, and they were mainly up-regulated in roots at 16 days post-inoculation (Supplementary Figure S2), indicating that these up-regulated C2H2-ZF genes (mainly including Gm-t1-SF, Gm-1i-Q-SF, Gm-1i-M-SF, and Gm-3i-SF genes) might play roles in early nodule development. Besides, the RNA-Seq results of the soybean C2H2-ZFPs at five important developmental stages (branching stage, flowering stage, fruiting stage, pod stage, and harvest stage; Yuan et al., 2017) showed that nearly half of these genes had expression during middle or late nodule development. In particularly, three clustering branches of genes were expressed most abundantly in all or most of above-mentioned nodule samples, including 10 Gm-t1-SF genes, three Gm-1i-Q-SF genes, two Gm-1i-M-SF genes, and six Gm-2i-Q-SF genes. Besides, 11 Gm-t1-SF genes, five Gm-1i-Q-SF genes, four Gm-1i-M-SF genes, five Gm-2i-Q-SF genes, four Gm-2i-M-SF genes, and one Gm-3i-SF gene also exhibited high expression levels in these five nodule samples (Figure 6). Among them, about one-third genes had high expressions in all of the five nodule samples, while the rest two-thirds genes exhibited modified expressions in different nodules between these five important developmental stages, suggesting that these soybean C2H2-ZFPs played different roles in legume-rhizobium symbiosis. For example, the most abundant clustering branches of Gm-2i-Q-SF genes had higher expression in nodules at pod stage and harvest stage compared with other nodule samples (Figure 6C), indicating that these six Gm-2i-Q-SF genes played a role in nodule senescence.

### Verification of RNA-seq results by qPCR and the expression analysis of the ortholog C2H2 genes in *M. truncatula* and common bean

**Figure 7:**
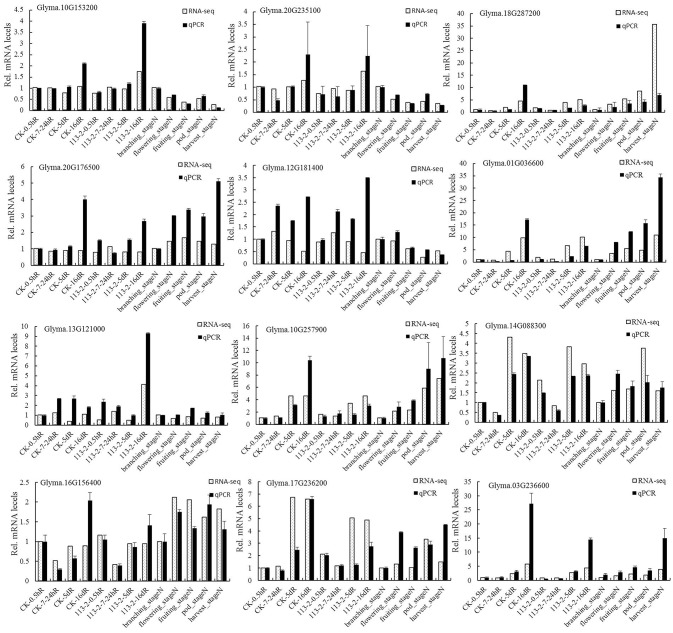
Comparison of expression determined by RNA-Seq and qPCR on 12 genes in soybean roots and nodule development. Half of the roots and nodules were inoculated with *B. japonicum* strain 113-2 and harvested at different time intervals post inoculation, and the other roots treated with water served as the uninoculation control. The relative expression levels of roots were calculated with reference to the value of the control roots harvested 0.5 h after mock treatment with water, and the relative expression levels of nodules were calculated with reference to the value of the branching_stage N. All qPCR reactions were repeated three times and the data are presented as the mean ± SD.

To verify the RNA-Seq results, the reference gene QACT (GmACT11, Glyma18g52780) was selected for the qPCR experiment according to our previous study (Yuan et al., 2017). The expressions of 12 soybean C2H2 genes that were randomly selected based on the RNA-Seq analysis were assessed by qPCR. The qPCR results were consistent with the RNA-Seq data for 133 out of 156 (85.3%) data points (Figure 7). Although the fold-changes were not exactly identical, both methods yielded identical expression trends for most data points. Supplementary Table S7 lists the sequences of the specific primers used for qPCR.

To confirm whether or not the potential candidate soybean C2H2 genes played roles in nodulation or nodule development, the ortholog genes of 44 selected soybean C2H2 genes in *M. truncatula* and common bean were identified, and their expressions in symbiosis were analyzed according to three databases (https://mtgea.noble.org/v3/index.php#__NO_LINK_PROXY__, http://www.phytozome.net and http://plantgrn.noble.org/PvGEA/Download.jsp) (Table 2, Supplementary Figure S3 and Supplementary Table S8). Two *M. truncatula* C2H2 genes had no expression data from *M. truncatula* Gene Expression Atlas database, while they had expression in nodules.symbiotic condition and/or roots. symbiotic condition according to the phytozome database (Table 2). The results showed that most of these ortholog genes had high expressions in symbiosis (different roots and nodules) or modified expressions during nodule development, or had roles in nodule formation (Table 2), indicating that these candidate soybean C2H2 genes might be indeed regulated or have a role in nodule development and/or nodule function.

**Table 2:** List of symbiosis-related C2H2 genes in *Medicago truncatula* and common soybean by searching for homologs of soybean.

**Soybean**	**Expression in symbiosis**	***M. truncatula***	**Expression in symbiosis**	**Common soybean**	**Expression in symbiosis**
Glyma.01G056900	Nodule development	Medtr5g043930	Nodule development	Phvul.002G082200	Nodule formation and nodule function
Glyma.01G079900	Nodule development	Medtr0160s0030	High expressed in nodules.symbiotic condition	Phvul.008G143900	Nodule development and nodule function
Glyma.01G134200	Nodule development	0		Phvul.010G071300	Nodule development and nodule function
Glyma.02G099100	Nodule development	Medtr1g093095	Nodule development	Phvul.003G027900	No expression in roots and nodules
Glyma.02G144400	Nodule development	0		Phvul.007G174900	Nodule development
Glyma.03G173100	Nodule development	0		Phvul.005G046200	Expressed in roots and nodules
Glyma.03G228100	Nodule development	Medtr7g111520	Nodule development	Phvul.001G222000	Nodule development
Glyma.04G044900,Glyma.06G045400	Nodule senescence	Medtr3g102980	High expressed in different nodules and roots	Phvul.009G070800	Nodule formation
Glyma.04G130300,Glyma.06G316400	High expressed in different nodules and roots	Medtr3g007710	Nodule development	Phvul.005G011500	Nodule formation
Glyma.04G191900	Nodule development	Medtr3g075210	High expressed in different nodules	Phvul.009G169700	Nodule formation
Glyma.05G241800,Glyma.08G049300	High expressed in different nodules and roots	Medtr8g106220	Nodule development	Phvul.002G325100	Nodule formation
Glyma.06G314900	High expressed in different nodules and roots	Medtr3g006760	High expressed in different nodules	Phvul.005G010800	Nodule development or formation
Glyma.07G135800,Glyma.18G185900	High expressed in different nodules	0		Phvul.008G117400	Nodule development and nodule function
Glyma.07G158200	Nodule development	0		Phvul.003G008300	Nodule formation
Glyma.09G250600,Glyma.18G241900	High expressed in different nodules and roots	0		Phvul.008G054200	Nodule development or formation
Glyma.09G267800	Nodule development	Medtr7g075170	Nodule development	Phvul.008G075100	Nodule development or formation
Glyma.10G034700,Glyma.13G121000	High expressed in different nodules	Medtr1g070250	Nodule development	Phvul.007G195500	Nodule development and nodule function
Glyma.10G055200	High expressed in different nodules and roots	Medtr1g063190	Expressed in nodules/roots.symbiotic condition	Phvul.007G219000	Nodule development or formation
Glyma.10G153200,Glyma.20G235100	Nodule development	0		Phvul.007G196100	Nodule formation
Glyma.10G184500,Glyma.20G206000	Nodule development	0		Phvul.007G125400	Nodule development and nodule function
Glyma.10G215200,Glyma.20G176500	High expressed in different nodules and roots	0		Phvul.007G090500	Nodule formation
Glyma.10G257900,Glyma.20G133200	Nodule senescence	Medtr1g106730	High expressed in different nodules and roots	Phvul.007G044500	Nodule development or formation
Glyma.11G135800,Glyma.12G059500	Nodule development	0		Phvul.011G061500	Nodule development and nodule function
Glyma.12G171700,Glyma.13G327500	High expressed in different nodules	0		Phvul.005G136300	Nodule formation
Glyma.12G183900,Glyma.13G317200	Nodule development	0		Phvul.005G124800	Nodule formation
Glyma.13G250800,Glyma.15G063600	Nodule development	Medtr2g010860	High expressed in different roots	Phvul.006G196000	Mainly expressed in nodules
Glyma.14G088300	Nodule senescence	Medtr1g018420	High expressed in different nodules	Phvul.001G026700	High expressed in nodules and roots
Glyma.16G156400	High expressed in different nodules and roots	0		Phvul.004G084300	Nodule formation
Glyma.18G186000	Nodule development	0		Phvul.008G117300	Nodule development or formation
Glyma.U019400	High expressed in different nodules and roots	Medtr4g059870	High expressed in different nodules	Phvul.011G074100	Nodule formation

*0″ indicates no match to the genome. The expression of soybean C2H2 in symbiosis was referred to Figure 6 and Supplementary Figure S2, the expression of Medicago truncatula C2H2 in symbiosis was referred to Supplementary Figure S4, and the expression of common soybean C2H2 was referred to Supplementary Table S8. The Soybean Genome Database [http://soybase.org/], Phytozome Database [http://www.phytozome.net/soybean], and NCBI-BLAST [http://blast.ncbi.nlm.nih.gov/] online resources were searched to identify the homologies*.

### Interaction and regulatory networks prediction of symbiosis-related soybean C2H2-ZFPs

To predict the interaction network of symbiosis-related soybean C2H2-ZFPs in legume-rhizobia symbiosis, we identify the predicted functional partners of the 26 selected soybean C2H2-ZFPs through SMART database, including the three clustering branches of genes with most abundant expression in nodules (Figure 6) and most genes of the specifically expressed branch (Figure 5B). Supplementary Figure S4 shows the interaction network prediction results with gene IDs in Wm82.a1.V1 version, and the corresponding Wm82.a2.V1 gene IDs of these old version genes were shown in Supplementary Table S9. Among these 26 C2H2-ZFPs, six Gm-1i-Q-SF genes did not have predicted interacting proteins, the other 20 members were divided into nine groups (with similarly interaction network), and the detailed information was shown in Table 3.

**Table 3:** The interacting protein prediction of symbiosis-related soybean C2H2-ZFPs.

**Groups**	**Symbiosis-related C2H2-ZFPs**	**Subsets**	**Predicted interactions network members**	**Annotations**
**a**	Glyma.02G144400	Gm-t1-SF	Glyma.05g189400, Glyma.08g147000,	Ran GTPase-activating protein
	Glyma.10G153200	Gm-t1-SF	Glyma.01g146600, Glyma.09g194200	cGMP-dependent protein kinase
	Glyma.12G171700	Gm-t1-SF	Glyma.07g249700, Glyma.09g046800, Glyma.15g154300	5′-AMP-activated protein kinase
	Glyma.20G235100	Gm-t1-SF	Glyma.17g024700, Glyma.19g234600, Glyma.20g234600	5′-AMP-activated protein kinase
	Glyma.U019400	Gm-t1-SF		
**b**	Glyma.16G156400	Gm-t1-SF	Glyma.05g189400, Glyma.08g147000	Ran GTPase-activating protein
	Glyma.10G215200 (STOP1)	Gm-t1-SF	Glyma.01g146600, Glyma.09g194200	cGMP-dependent protein kinase
			Glyma.01g199400,Glyma.11g042500, Glyma.17g154200	Predicted membrane protein
			Glyma.10g047100	Putative ABC transport system permease protein
			Glyma.20g234600	5′-AMP-activated protein kinase
**c**	Glyma.13G327500	Gm-t1-SF	Glyma.05g189400, Glyma.08g147000	Ran GTPase-activating protein
			Glyma.01g146600, Glyma.09g194200	cGMP-dependent protein kinase
			Glyma.10g082800	CCAAT-binding transcription factor
			Glyma.01g008700	RNA polymerase
			Glyma.14g110900	C2H2-Zinc finger
			Glyma.17g065800	Myb-like transcription factor
			Glyma.14G050100	ERF5
			Glyma.20g234600	5′-AMP-activated protein kinase
**d**	Glyma.05G241800	Gm-t1-SF	Glyma.02g297300, Glyma.08g325700,Glyma.18g081400,	Frigida-like protein
	Glyma.08G049300	Gm-t1-SF	Glyma.04g203400, Glyma.06g162100	Frigida-like protein
			Glyma.10g258300, Glyma.20g132800,Glyma.20g133400,	mRNA export factor and BUB3
			Glyma.02g112500, Glyma.10g257700	mRNA export factor and BUB3
**e**	Glyma.18G241900	Gm-1i-M-SF	Glyma.02g187100, Glyma.10g106100	Translation initiation factor 3 subunit E
	Glyma.09G250600	Gm-1i-M-SF	Glyma.09g227200, Glyma.12g009600	Large subunit ribosomal protein L1
			Glyma.10g055200,Glyma.13g142300	C2H2-Zinc finger (Gm-1i-M-SF)
			Glyma.13G333200	MYB139
			Glyma.15g101700	F-box protein
			Glyma.18g012400	TCP-1/cpn60 chaperonin family
**f**	Glyma.04G044900	Gm-2i-Q-SF	Glyma.07g023300,Glyma.08g218600, Glyma.13g370100, Glyma.15g003300	WRKY56, WRKY78, WRKY transcription factor 40- related
	Glyma.06G045400 (SCTF-1)	Gm-2i-Q-SF	Glyma.02g138800, Glyma.07g206200,Glyma.12g073000, Glyma.U021800	MAPK2, MAPK1, MAPK
	Glyma.14G088300	Gm-2i-Q-SF	Glyma.01g146600,Glyma.09g194200,	cGMP-dependent protein kinase
**g**	Glyma.17G236200 (SCOF-1)	Gm-2i-Q-SF	Glyma.07g023300,Glyma.08g218600, Glyma.13g370100, Glyma.15g003300	WRKY56, WRKY78, WRKY transcription factor 40- related
			Glyma.12g073000, Glyma.07g206200, Glyma.U021800	MAPK1, MAPK
			Glyma.01g146600, Glyma.09g194200	cGMP-dependent protein kinase
			Glyma.10g013300	BZIP59
**h**	Glyma.10G257900	Gm-2i-Q-SF	Glyma.01g146600, Glyma.09g194200	cGMP-dependent protein kinase
	Glyma.20G133200	Gm-2i-Q-SF	Glyma.15g154300, Glyma.17g024700, Glyma.19g234600, Glyma.20g234600	5′-AMP-activated protein kinase
			Glyma.05g189400, Glyma.08g147000	Ran GTPase-activating protein
			Glyma.12g179000	C2H2-Zinc finger (Gm-t1-SF)
**i**	Glyma.12G059500	Gm-1i-Q-SF	Glyma.18G124800	Whirly transcription factor
	Glyma.11G135800	Gm-1i-Q-SF		
	Glyma.10G184500	Gm-1i-Q-SF	No	
	Glyma.07G135800 (GmRSD2)	Gm-1i-Q-SF	No	
	Glyma.20G206000	Gm-1i-Q-SF	No	
	Glyma.18G185900 (GmRSD1)	Gm-1i-Q-SF	No	
	Glyma.10G034700	Gm-1i-Q-SF	No	
	Glyma.13G121000	Gm-1i-Q-SF	No	

**Figure 8:**
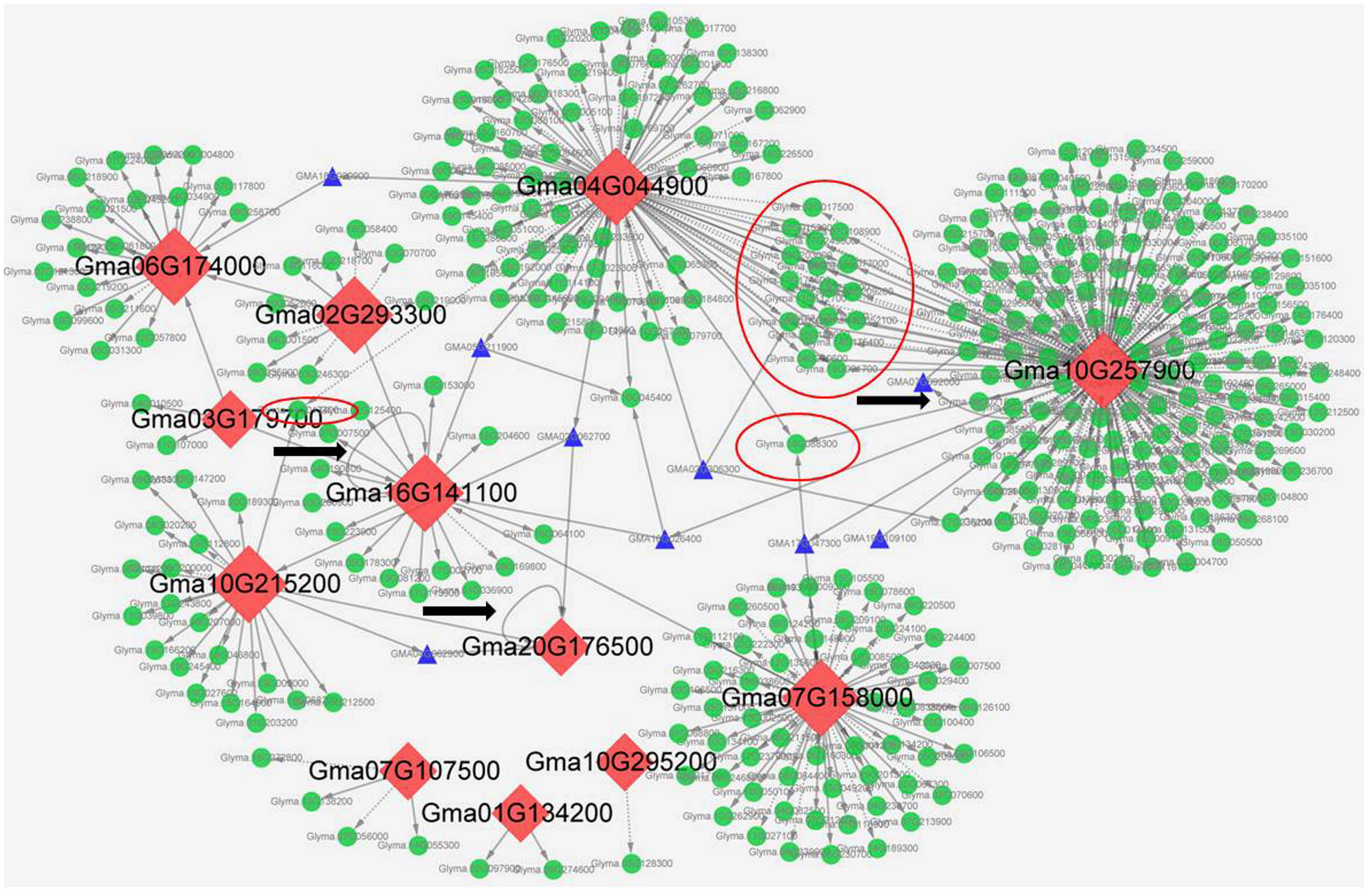
The transcriptional regulatory networks of 12 symbiosis-related soybean C2H2-TFs. These transcriptional regulatory networks were constructed by Cytoscape software. The red rhombuses represent C2H2-TFs, the green circles pointed by arrows represent the regulated downstream genes, blue triangle pointed by arrows represent the upstream genes or bridge genes, the red circle represents the co-regulated genes, and the gray ring arrow represents the self-regulation of C2H2-TFs. Besides, the solid arrows indicate the up-regulation genes, and the dotted arrows indicate the down-regulation genes.

To predict the genes regulated by symbiosis-related C2H2-ZFPs, we performed transcriptional regulatory network analysis of the 12 selected C2H2-ZFPs through plant transcriptional regulatory map database (http://plantregmap.cbi.pku.edu.cn/download.php#networks), and our previous RNA-seq data (Yuan et al., 2016a, 2017). The transcriptional regulatory network prediction results showed that Glyma.10G257900 had the most regulatory genes, followed by Glyma.04G044900 and Glyma.07G158000. Some of the 12 C2H2-ZFPs had identical regulatory genes. For example, Glyma.04G044900 and Glyma.10G257900 had about 20 identical regulatory genes. It was worth noting that self - regulation was observed in Glyma.16G141100 and Glyma.20G176500, while Glyma.10G257900 regulated itself through the other gene (Glyma.07G092000). Besides, some C2H2-ZFPs could regulate other C2H2-ZFPs through other bridge genes. For example, Glyma.10G215200 could regulate Glyma.20G176500 through Glyma.04G062900, Glyma.04G044900 could regulate Glyma.06G174000 through Glyma.18G20900, Glyma.04G044900 could regulate Glyma.16G141100 through Glyma.05G211900, and so on (Figure 8). Figure 8 shows the detailed upstream and downstream regulatory genes and the cross characteristics between these C2H2-ZFPs. Supplementary Table S10 illustrates the detailed information of regulatory genes.

## Discussion

The symbiotic nitrogen fixation of legumes can reduce the application of nitrogen fertilizer and is of great significance to the development of environment-friendly green agriculture (Biswas and Gresshoff, 2014). Previous studies have shown that C2H2-ZF genes play important roles in symbiosis establishment, normal nodule development and symbiotic nitrogen fixation (Sinharoy et al., 2013; Diedhiou et al., 2014). However, a great variation of structure and function exist among the family members. It remains unclear whether or not special types of C2H2-ZF genes participate in symbiosis, especially in soybean. To determine which types of soybean C2H2-ZFPs were involved in legume-rhizobium symbiosis, we, for the first time, classified the soybean C2H2-ZFPs and characterized the sequences and GO functions of these clearly distinguishable subsets. Then the genome-wide survey of soybean C2H2-ZFPs in nodule symbiosis was conducted, and the resultant expression patterns of different types of C2H2-ZFPs in nodulation and nodule development were used to explore their putative roles in nodule symbiosis. The predicted functional partners and regulatory networks of symbiosis-related soybean C2H2-ZFPs provided useful information or clues for our understanding of the roles in symbiosis of C2H2-ZFPs in soybean or other legumes.

### Identification and classification of soybean C2H2-ZFPs

Genome-wide analysis of the C2H2-ZF gene family has been performed in several plants (Englbrecht et al., 2004; Agarwal et al., 2007; Muthamilarasan et al., 2014; Liu et al., 2015; Minglei et al., 2016). However, this family has not been previously studied in legume, especially in soybean. In this report, we identified 321 full-length C2H2-ZFPs (about 0.69% of all the soybean protein-coding genes; Schmutz et al., 2010) in soybean genome (Supplementary Table S1 and Figure 1). Besides, previous studies have revealed that the Glycine-specific WGD event (13 Ma) significantly contributes to the expansion of the soybean genome (Egan and Doyle, 2010; Schmutz et al., 2010; Cannon et al., 2015), and 15,632 gene pairs and 15,166 singletons exist in soybean genome after the WGD event (Schmutz et al., 2010). Here, the expansion of soybean C2H2-ZFPs was also mainly caused by the Glycine-specific WGD event, and about 92.2% of soybean C2H2-ZFPs (296 of 321) were preferentially retained duplicates, including 135 pairs and 22 single genes (Figure 1 and Supplementary Table S1). These identified soybean C2H2-ZFPs encoded peptides with 87 to 1,592 amino acid residues, and the locations, numbers and types of the C2H2 domain significantly varied among these C2H2-ZFPs (Supplementary Tables S1, S4). These findings indicated that a high degree of complexity existed in soybean C2H2-ZF gene family, which was consistent with the low bootstrap supports of some main branches in phylogenetic tree analysis (Figure 3 and Supplementary Figure S1).

C2H2-ZFPs have been classified into a few main subsets in yeast and *Arabidopsis* based on the numbers, types and arrangements of their ZF domains (Bohm et al., 1997; Englbrecht et al., 2004). In plants, the different types of C2H2-ZFPs have been defined based on the modification of the conserved motif “QALGGH” (Takatsuji, 1999; Liu et al., 2015; Zhang et al., 2016). In the present study, soybean C2H2-ZFPs were classified into 11 subsets based on the arrangements, numbers, and types of C2H2-ZF domains (Figure 2, Supplementary Tables S3, S4). Many studies on C2H2-ZFPs have demonstrated that the molecular functions of the proteins with tandem C2H2-ZF domain are usually different from the C2H2-ZFPs with dispersed fingers (Bohm et al., 1997; Wolfe et al., 2000; Englbrecht et al., 2004; Agarwal et al., 2007; Xiong et al., 2015). Therefore, the proteins with single or dispersed C2H2-ZF domains are usually categorized separately from tandem C2H2-ZF domain proteins (Englbrecht et al., 2004; Xiong et al., 2015). In this report, soybeans C2H2-ZFPs were also classified into two Gmtandem-SF subsets and nine Gmisolated-SF subsets (Figure 2 and Supplementary Table S4). Based on the statistical analysis results of linker lengths in soybean C2H2-ZFPs (Supplementary Table S4), C2H2-ZFPs with two or more fingers and every two adjacent domains linked by <12 amino acid residues were defined as tandem ZFs, which is different from the tandem ZFs in yeast and *Arabidopsis* (Bohm et al., 1997; Englbrecht et al., 2004). Gm-t1-SF and Gm-t2-SF subsets were defined based on the numbers of C2H2-ZF domains according to the Set A and Set B in *Arabidopsis* C2H2-ZFPs (Englbrecht et al., 2004). Moreover, like yeast and animal, there was no Q-type C2H2-ZF domain in these C2H2-ZFPs (Supplementary Table S4). *Arabidopsis* C2H2-ZFPs with isolated C2H2-ZF domains have been classified into three main subsets (C1, C2, and C3) based on the spacing between the two invariant zinc coordinating histidine residues, and these three main subsets are further divided into several categories according to all of the domains in C2H2-ZFPs (including non-finger domains; Englbrecht et al., 2004). In soybean, Gmisolated-SF was further classified into nine clearly distinguishable subsets (Gm-1i-Q-SF, Gm-1i-M-SF, Gm-1i-Z-SF, Gm-1i-D-SF, Gm-2i-Q-SF, Gm-2i-M-SF, Gm-2i-Mix-SF, Gm-3i-SF, Gm-4i-SF) based on the numbers and types of C2H2-ZF domains (Figure 2 and Supplementary Table S4). Besides, four types of C2H2-ZF domains (Q-type, M-type, Z-type, and D-type) in soybean were defined (Supplementary Table S3), and the Q-type and M-type C2H2 ZF domains were similar, but not the same, to those defined in previous studies (Muthamilarasan et al., 2014; Liu et al., 2015). The rest two types were different from the types defined in other reports, the Z-type domain was defined when the spacing between the second cysteine and the first histidine was <12 amino acids, and the D-type did not include the second histidine in the C2H2-ZF domain (Supplementary Table S3).

To evaluate the 11 classified subsets, we performed phylogenetic and GO analyses to examine the conserved sequence and Go function among these subsets (Figures 3, 4). Most of C2H2-ZFPs in the same subset was clustered together and had more than 50% bootstrap support (Figure 3 and Supplementary Figure S1), and more than 65.6% Go slim terms had only one subset of C2H2-ZFPs (Table 1). These findings indicated that the sequences and biological functions were relatively conservative among the classified 11 subsets of C2H2-ZFPs, and the classification of soybean C2H2-ZFPs was reasonable.

### Potential roles of different subsets of soybean C2H2-ZFPs in legume-rhizobium symbiosis

Previous studies have shown that some C2H2-ZF genes play important roles in symbiosis (Sinharoy et al., 2013; Diedhiou et al., 2014). C2H2-ZFPs are the most widespread ZFPs in eukaryotes, and a great variation of structure and function exist among the family members (Tadepally et al., 2008; Cao et al., 2016). To determine which types of soybean C2H2-ZFPs played roles in nodulation and/or nodule development, we first assessed the expression profiles of 11 clearly distinguishable subsets of C2H2-ZFPs in various soybean tissues, and the results showed that nearly half of soybean C2H2-ZFPs within different subsets had expressions in nodules (Figure 5). Next, we analyzed the expression profiles of these 11 classified subsets of C2H2-ZFPs in roots and nodules at different developmental stages by RNA-Seq (Figure 6 and Supplementary Figure S2), which is an effective method with greater sensitivity, higher reproducibility and wider dynamic range compared with other conventional methods (Marioni et al., 2008). Thirdly, we performed qPCR experiment and expression analysis of the ortholog C2H2 genes in *M. truncatula* and common bean (Figure 7, Table 2, Supplementary Figure S3 and Supplementary Table S8) to verify the RNA-Seq data. Our qPCR results were consistent with the transcriptional profile data for 133 out of 156 (85.3%) data points (Figure 7), and most of the ortholog C2H2 genes in *M. truncatula* and common bean had expression in symbiosis or played roles in nodule formation, nodule development or nodule function (Table 2, Supplementary Figure S3 and Supplementary Table S8), suggesting that our RNA-Seq data were reliable.

In the present study, the RNA-Seq data showed that very few part of the soybean C2H2-ZFPs were up-regulated during root development after inoculation (Supplementary Figure S2), nearly half of these genes had expressions in all or most of the nodule samples, and they showed different expression patterns during soybean nodule development (Figure 6). Besides, among the 11 Gm-1i-Q-SF genes of the symbiosis-specific expression branch (Figure 5), only five C2H2-ZFPs (Glyma.07G135800, Glyma.10G034700, Glyma.18G185900, Glyma.12G059500, and Glyma.11G135800) were up-regulated in roots at 16 days post-inoculation (Supplementary Figure S2B), and they had relatively abundant expressions in different nodule samples (Figure 6B). These results indicated that soybean C2H2-ZFPs mainly played roles in nodule development or nodule function rather than nodulation signal transduction.

Nodule development directly affects efficiency of nitrogen fixation, which changes with the growth of soybean (Hardy et al., 1968), and our previous study has shown the nitrogen fixation characteristics of soybean at five important developmental stages (branching stage, flowering stage, fruiting stage, pod stage, and harvest stage; Yuan et al., 2017). In this study, nearly half of soybean C2H2-ZFPs showed different expression patterns in nodules during these five important developmental stages (Figure 6). It was worth noting that the six clustering branches of C2H2-ZF genes (including 21 Gm-t1-SF genes, eight Gm-1i-Q-SF genes, 11 Gm-2i-Q-SF genes, six Gm-1i-M-SF genes, four Gm-2i-M-SF genes, and one Gm-3i-SF gene) were expressed more abundantly in all or most of these nodule samples. Among them, two Gm-t1-SF genes (Glyma.16G156400 and Glyma.12G171700) and one Gm-3i-SF gene (Glyma.01G056900) showed highest expression at the stage with relatively higher nitrogen-fixation rate (flowering stage and fruiting stage), indicating that they played roles in the nitrogen-fixation process. The highest expressions of four Gm-t1-SF genes (Glyma.20G235100, Glyma.10G153200, Glyma.13G327500, and Glyma.02G144400) were detected in branching stage, indicating that they played roles in early or middle nodule development. For nodule senescence, our study mainly focused on the Gm-2i-Q-SF genes, and eight of these genes (Glyma.17G236200, Glyma.06G045400, Glyma.14G088300, Glyma.10G257900, Glyma.04G044900, Glyma.20G133200, Glyma.13G133100, and Glyma.15G040700) were up-regulated in nodules at the pod stage and/or harvest stage (Figure 6C). The specific regulatory roles and distinct functions of above-mentioned soybean C2H2-ZF genes were interesting and worthy to be explored in the future.

### Search of the interacting and/or regulated proteins of symbiosis-related soybean C2H2-ZFPs

Different proteins often form protein complex through complicated interactions to perform their biological functions (Morsy et al., 2008; Braun et al., 2013). C2H2-ZFPs can interact not only with themselves, but also with other types of proteins to form protein complex (Sinharoy et al., 2013; Rizkallah et al., 2015). According to the predicted interacting proteins, two of the Gm-t1-SF genes had different predicted interacting proteins from the other eight members (Table 3). This was mainly because that these two genes (Glyma.05G241800 and Glyma.08G049300) only had two C2H2-ZF domains, while the others had three C2H2-ZF domains in proteins (Supplementary Table S4). Four of the Gm-2i-Q-SF genes (Glyma.04G044900, Glyma.06G045400, Glyma.14G088300, and Glyma.17G236200) might form protein complex through interacting with WRKY transcription factors (TFs) and function through MAPK signaling pathway (Table 3). The other two genes (Glyma.10G257900 and Glyma.20G133200) could interact with a Gm-t1-SF gene (Glyma.12g179000) and had same predicted interacting proteins with groups a, b, and c (Table 3), indicating that these two genes might perform their biological functions through forming protein complex with Gm-t1-SF genes and the same interacting proteins.

Transcriptional regulatory networks of TFs have become more important and attractive to identify the target regulatory genes and interacting proteins of the TFs (Barabási and Oltvai, 2004; Jang et al., 2015). The regulatory mechanism of C2H2-ZFPs in legume-rhizobium symbiosis remains largely unexplored, especially in soybean. In this study, we performed transcriptional regulatory network analysis of soybean C2H2-ZFPs through plant transcriptional regulatory map database (http://plantregmap.cbi.pku.edu.cn/download.php#networks) and our previous symbiosis-related RNA-seq data (Yuan et al., 2016a, 2017), and finally selected 12 symbiosis-related C2H2-ZFPs (Figure 8). According to the predicted regulatory networks, Glyma.10G257900 and Glyma.04G044900 had relative more regulatory genes than other C2H2-ZFPs (Figure 8 and Supplementary Table S10), indicating that nodule senescence was a very complex process and involved in a lot of regulatory pathways. Besides, C2H2-ZFPs could not only interact with themselves (Table 3), but also regulate themselves directly or indirectly (Figure 8).

The interaction and regulatory networks analysis of these genes showed that nearly no known symbiosis-related functional proteins in these predicted interacting or regulated proteins (Table 3 and Supplementary Table S10), indicating that these soybean C2H2-ZFPs functioned in legume-rhizobium symbiosis through regulating or interacting with other key proteins. These networks analysis could provide useful information for the study on nodule function or nodule development and senescence.

In summary, we aimed to classify the soybean C2H2-ZFPs and identify the symbiosis-related types of C2H2-ZFPs in soybean in the present study. A total of 321 C2H2-ZFPs were identified in soybean genome through a genome-wide survey. The detailed gene information showed a high degree of complexity in soybean C2H2-ZF gene family. Phylogenetic and GO analyses of the 11 classified soybean C2H2-ZFPs indicated the classification reasonableness of soybean C2H2-ZFPs in this report. Expression profiles of C2H2-ZFPs in different soybean tissues and symbiosis developmental stages were used to explore symbiosis-related C2H2-ZFPs. The interaction and regulatory network analysis showed that the symbiosis-related C2H2-ZFPs functioned in legume-rhizobium symbiosis through regulating or interacting with other key proteins. Our findings provided valuable evidence for rhizobium symbiosis-related C2H2 ZFPs in soybean, and offered useful information for the classification of plant C2H2-ZFPs.

## Author contributions

SY and XinZ: designed this work; SY: wrote the manuscript; SY, XL, and RL: performed most of the experiments and analysis; LW, CZ, LC, QH, XiaZ, HC, ZS, ZY, SC, DQ, and DK: contributed substantially to the completion of this work; All authors read and approved the final manuscript.

### Conflict of interest statement

The authors declare that the research was conducted in the absence of any commercial or financial relationships that could be construed as a potential conflict of interest.

## Supplementary material

Supplementary Table S1 The detailed information of soybean C2H2-ZFPs.

Supplementary Table S2 The ID information of the 82 soybean C2H2-ZFPs that not in PlantTFDB.

Supplementary Table S3 Types of C2H2-ZF domains in soybean C2H2-ZFPs.

Supplementary Table S4 Complete set of the classified C2H2-ZFPs in soybean.

Supplementary Table S5 The gene ID of the 17 GO function terms in each subset of soybean C2H2-ZFPs.

Supplementary Table S6 The gene ID information of the 32 GO slim terms in soybean C2H2-ZFPs.

Supplementary Table S7 Primers for genes selected ford qPCR analysis.

Supplementary Table S8 Expression analysis of some common soybean C2H2-ZFPs in different roots and nodules.

Supplementary Table S9 ID information of symbiosis-related C2H2-ZFPs and their interaction proteins.

Supplementary Table S10 The detail gene information of the transcriptional regulatory networks of 12 symbiosis-related soybean C2H2-TFs.

Supplementary Figure S1 The detailed gene ID information in phylogenetic analysis of the 11 classified soybean C2H2-ZFP subsets.

Supplementary Figure S2 RNA-seq analysis of 321 soybean C2H2-ZFPs in soybean roots at different time points.

Supplementary Figure S3 The Expression analysis of *M. truncatula* C2H2-ZFPs in different root hairs and different nodules.

Supplementary Figure S4 The interaction network prediction results of the selected symbiosis-related soybean C2H2-ZFPs.
