# Supplementary material for: Genome-Wide Identification and Classification of Soybean C2H2 Zinc Finger Proteins and Their Expression Analysis in Legume-Rhizobium Symbiosis
Source: Front Microbiol. 2018 Feb 6;9:126. doi: 10.3389/fmicb.2018.00126 (PMC5807899; doi:10.3389/fmicb.2018.00126)
Supplement: Supplementary Table S2 — The ID information of the 82 soybean C2H2-ZFPs that not in PlantTFDB. [file Table2.docx]

**Table S2: The ID information of the 82 soybean C2H2**-**ZFPs that not in PlantTFDB.**

| **No.** | **Gene ID** |
| --- | --- |
| 1 | Glyma.01G056900 |
| 2 | Glyma.01G134100 |
| 3 | Glyma.01G180100 |
| 4 | Glyma.01G206000 |
| 5 | Glyma.02G060500 |
| 6 | Glyma.03G033700 |
| 7 | Glyma.03G147200 |
| 8 | Glyma.03G160900 |
| 9 | Glyma.03G222900 |
| 10 | Glyma.03G231900 |
| 11 | Glyma.04G128800 |
| 12 | Glyma.04G133900 |
| 13 | Glyma.04G164300 |
| 14 | Glyma.04G236800 |
| 15 | Glyma.04G252600 |
| 16 | Glyma.05G021300 |
| 17 | Glyma.05G154200 |
| 18 | Glyma.05G241800 |
| 19 | Glyma.06G109900 |
| 20 | Glyma.06G127500 |
| 21 | Glyma.06G201200 |
| 22 | Glyma.06G265800 |
| 23 | Glyma.06G314900 |
| 24 | Glyma.07G144300 |
| 25 | Glyma.08G049300 |
| 26 | Glyma.08G112000 |
| 27 | Glyma.08G178400 |
| 28 | Glyma.08G227300 |
| 29 | Glyma.08G242800 |
| 30 | Glyma.08G319000 |
| 31 | Glyma.08G356600 |
| 32 | Glyma.08G366900 |
| 33 | Glyma.09G107400 |
| 34 | Glyma.09G250600 |
| 35 | Glyma.09G267800 |
| 36 | Glyma.10G034700 |
| 37 | Glyma.10G055200 |
| 38 | Glyma.10G118800 |
| 39 | Glyma.10G272300 |
| 40 | Glyma.10G273800 |
| 41 | Glyma.11G036800 |
| 42 | Glyma.11G084900 |
| 43 | Glyma.11G157700 |
| 44 | Glyma.12G136900 |
| 45 | Glyma.12G215700 |
| 46 | Glyma.13G073300 |
| 47 | Glyma.13G086300 |
| 48 | Glyma.13G107200 |
| 49 | Glyma.13G142300 |
| 50 | Glyma.13G250800 |
| 51 | Glyma.13G285800 |
| 52 | Glyma.13G340800 |
| 53 | Glyma.13G367400 |
| 54 | Glyma.14G084900 |
| 55 | Glyma.15G005900 |
| 56 | Glyma.15G063600 |
| 57 | Glyma.16G051400 |
| 58 | Glyma.16G143100 |
| 59 | Glyma.17G052200 |
| 60 | Glyma.17G078100 |
| 61 | Glyma.17G189600 |
| 62 | Glyma.17G193300 |
| 63 | Glyma.17G200200 |
| 64 | Glyma.18G094500 |
| 65 | Glyma.18G174500 |
| 66 | Glyma.18G195500 |
| 67 | Glyma.18G222100 |
| 68 | Glyma.18G241900 |
| 69 | Glyma.18G265000 |
| 70 | Glyma.18G295500 |
| 71 | Glyma.19G070000 |
| 72 | Glyma.19G097200 |
| 73 | Glyma.19G150800 |
| 74 | Glyma.19G162600 |
| 75 | Glyma.19G191000 |
| 76 | Glyma.19G220000 |
| 77 | Glyma.19G228900 |
| 78 | Glyma.20G069800 |
| 79 | Glyma.20G116200 |
| 80 | Glyma.20G117800 |
| 81 | Glyma.U011900 |
| 82 | Glyma.U012000 |
