# Supplementary material for: Genome-Wide Identification and Classification of Soybean C2H2 Zinc Finger Proteins and Their Expression Analysis in Legume-Rhizobium Symbiosis
Source: Front Microbiol. 2018 Feb 6;9:126. doi: 10.3389/fmicb.2018.00126 (PMC5807899; doi:10.3389/fmicb.2018.00126)
Supplement: Supplementary Table S3 — Types of C2H2-ZF domains in soybean C2H2-ZFPs. [file Table3.docx]

**Table S3: Types of C2H2-ZF domains in soybean C2H2-ZFPs.**

| **Type of ZF** | | **Conserved motif sequence description** | **Conserved spacing^^^** |
| --- | --- | --- | --- |
| **Q** |  | QALGGH | X2-C-X2-C-X7-QALGGH-X3-H |
| **M** | **M1** | 1 Degraded amino acids in QALGGH | X2-C-X2-C-X12-H-X(3,4)-H |
|  | **M2** | 2 Degraded amino acids in QALGGH | X2-C-X2-C-X12-H-X(3,4)-H |
|  | **M3** | 3 Degraded amino acids in QALGGH | X2-C-X(2,4)-C-X12-H-X(3,4,5)-H |
|  | **M4** | 4 Degraded amino acids in QALGGH | X2-C-X(1,2,4)-C-X12-H-X(1,3,4,5,7)-H |
|  | **M5** | 5 Degraded amino acids in QALGGH | X2-C-X(2,4)-C-X12-H-X(3,4,5,8)-H |
| **Z** | **Z1** |  | X2-C-X(2,3,4)-C-X(>12)-H-X(2,3,4,5)-H |
|  | **Z2** |  | X2-C-X2-C-X(<12)-H-X(3,4)-H |
| **D** |  |  | X2-C-X(2,4)-C-X(12,13)-H-X2 |

^The number represents the consensus spacing between the conserved amino acid residues.
