# Supplementary material for: Genome-Wide Identification and Classification of Soybean C2H2 Zinc Finger Proteins and Their Expression Analysis in Legume-Rhizobium Symbiosis
Source: Front Microbiol. 2018 Feb 6;9:126. doi: 10.3389/fmicb.2018.00126 (PMC5807899; doi:10.3389/fmicb.2018.00126)
Supplement: Supplementary Table S9 — ID information of symbiosis-related C2H2-ZFPs and their interaction proteins. [file Table9.docx]

**Table S9: ID information of symbiosis**-**related C2H2**-**ZFPs and their interaction proteins.**

| Symbiosis-related C2H2-ZFPs | | Predicted interactions network members | |
| --- | --- | --- | --- |
| Wm82.a1.V1 | Wm82.a2.V1 | Wm82.a1.V1 | Wm82.a2.V1 |
| Glyma02g16280 | Glyma.02G144400 | Glyma01G01210 | Glyma.01g008700 |
| Glyma10g29390 | Glyma.10G153200 | Glyma01G34840 | Glyma.01g146600 |
| Glyma12g29370 | Glyma.12G171700 | Glyma01G40760 | Glyma.01g199400 |
| Glyma20g37900 | Glyma.20G235100 | Glyma02G12530 | Glyma.02g112500 |
| Glyma11G15950 | Glyma.U019400 | MAPK2(Glyma02g15690) | Glyma.02g138800 |
| Glyma10g40400 | Glyma.10G257900 | Glyma02G33770 | Glyma.02g187100 |
| Glyma20g26940 | Glyma.20G133200 | Glyma02G46680 | Glyma.02g297300 |
| Glyma16g27280 | Glyma.16G156400 | Glyam03G33290 | No |
| STOP1(Glyma10g35940) | Glyma.10G215200 | Glyma04G38071 | Glyma.04g203400 |
| Glyma13g40240 | Glyma.13G327500 | Glyma05G32290 | Glyma.05g189400 |
| Glyma18g47731 | Glyma.18G241900 | Glyma06G17005 | Glyma.06g162100 |
| Glyma09g38590 | Glyma.09G250600 | Glyma07G02630 | Glyma.07g023300 |
| Glyma12g06340 | Glyma.12G059500 | Glyma07G32750 | Glyma.07g206200 |
| Glyma11g14400 | Glyma.11G135800 | Glyma07G37950 | Glyma.07g249700 |
| Glyma05g34280 | Glyma.05G241800 | Glyma08G15560 | Glyma.08g147000 |
| Glyma08g05390 | Glyma.08G049300 | WRKY56(Glyma08g23380) | Glyma.08g218600 |
| Glyma04g04760 | Glyma.04G044900 | Glyma08G40850 | No |
| SCTF-1(Glyma06g04840) | Glyma.06G045400 | Glyma08G43760 | Glyma.08g325700 |
| STF-3(Glyma14g09760) | Glyma.14G088300 | Glyma09G05190 | Glyma.09g046800 |
| SCOF-1(Glyma17g35430) | Glyma.17G236200 | Glyma09G32680 | Glyma.09g194200 |
|  |  | Glyma09G36120 | Glyma.09g227200 |
|  |  | BZIP59(Glyma10g01640) | Glyma.10g013300 |
|  |  | Glyma10G05420 | Glyma.10g047100 |
|  |  | Glyma10G06240 | Glyma.10g055200 |
|  |  | Glyma10G10240 | Glyma.10g082800 |
|  |  | Glyma10G16090 | Glyma.10g106100 |
|  |  | Glyma10G40380 | Glyma.10g257700 |
|  |  | Glyma10G40440 | Glyma.10g258300 |
|  |  | Glyma11G04570 | Glyma.11g042500 |
|  |  | MAPK1(Glyma11g15700) | Glyma.U021800 |
|  |  | Glyma12G01210 | Glyma.12g009600 |
|  |  | Glyma12G07770 | Glyma.12g073000 |
|  |  | Glyma12G30285 | Glyma.12g179000 |
|  |  | Glyma13G20550 | Glyma.13g142300 |
|  |  | Glyma13G39610 | No |
|  |  | MYB139(Glyma13g40830) | Glyma.13G333200 |
|  |  | Glyma13G44730 | Glyma.13g370100 |
|  |  | ERF5(Glyma14g05470) | Glyma.14G050100 |
|  |  | Glyma14G13360 | Glyma.14g110900 |
|  |  | LOC732588(Glyma15g00570,WRKY78 ) | Glyma.15g003300 |
|  |  | Glyma15G04620 | No |
|  |  | Glyma15G10860 | Glyma.15g101700 |
|  |  | Glyma15G16510 | Glyma.15g154300 |
|  |  | Glyma17G02750 | Glyma.17g024700 |
|  |  | Glyma17G07330 | Glyma.17g065800 |
|  |  | Glyma17G16520 | Glyma.17g154200 |
|  |  | Glyma18G01580 | Glyma.18g012400 |
|  |  | Glyma18G09060 | Glyma.18g081400 |
|  |  | Glyma18G16200 | Glyma.18G124800 |
|  |  | Glyma19G42290 | Glyma.19g234600 |
|  |  | Glyma20G26890 | Glyma.20g132800 |
|  |  | Glyma20G26960 | Glyma.20g133400 |
|  |  | Glyma20G37840 | Glyma.20g234600 |
