# Supplementary figures and images for: Genome-Wide Identification and Classification of Soybean C2H2 Zinc Finger Proteins and Their Expression Analysis in Legume-Rhizobium Symbiosis
Source: Front Microbiol. 2018 Feb 6;9:126. doi: 10.3389/fmicb.2018.00126 (PMC5807899; doi:10.3389/fmicb.2018.00126)

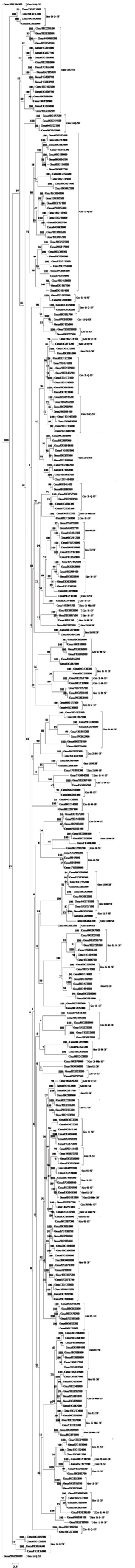

Supplement: Supplementary Figure S1 — The detailed gene ID information in phylogenetic analysis of the 11 classified soybean C2H2-ZFP subsets. [file Image1.pdf]
