# Supplementary material for: Genome-Wide Identification and Classification of Soybean C2H2 Zinc Finger Proteins and Their Expression Analysis in Legume-Rhizobium Symbiosis
Source: Front Microbiol. 2018 Feb 6;9:126. doi: 10.3389/fmicb.2018.00126 (PMC5807899; doi:10.3389/fmicb.2018.00126)
Supplement: Supplementary Figure S4 — The interaction network prediction results of the selected symbiosis-related soybean C2H2-ZFPs. [file Image4.pdf]

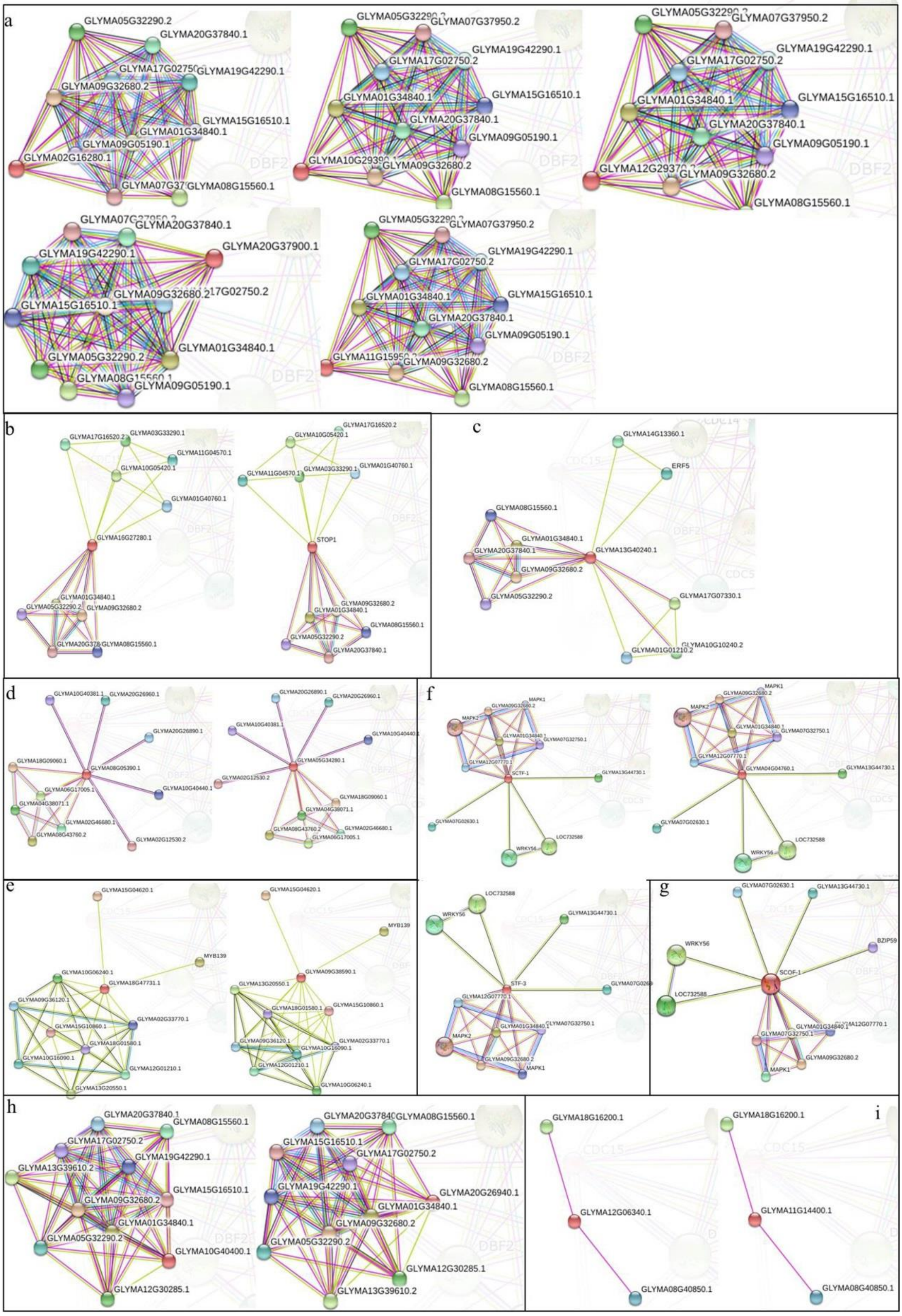

Supplemental Figure S4: The interaction network prediction results of the selected symbiosis-related soybean C2H2-ZFPs. This interaction network prediction analysis was done according to the SMART database, and the gene IDs in this interaction network prediction results is in Wm82.a1.V1 version. a to i represent nine groups (with similarly interaction network).
