# Supplementary material for: Genome-Wide Identification and Classification of Soybean C2H2 Zinc Finger Proteins and Their Expression Analysis in Legume-Rhizobium Symbiosis
Source: Front Microbiol. 2018 Feb 6;9:126. doi: 10.3389/fmicb.2018.00126 (PMC5807899; doi:10.3389/fmicb.2018.00126)
Supplement: Supplementary Figure S2 — RNA-seq analysis of 321 soybean C2H2-ZFPs in soybean roots at different time points. [file Image2.pdf]

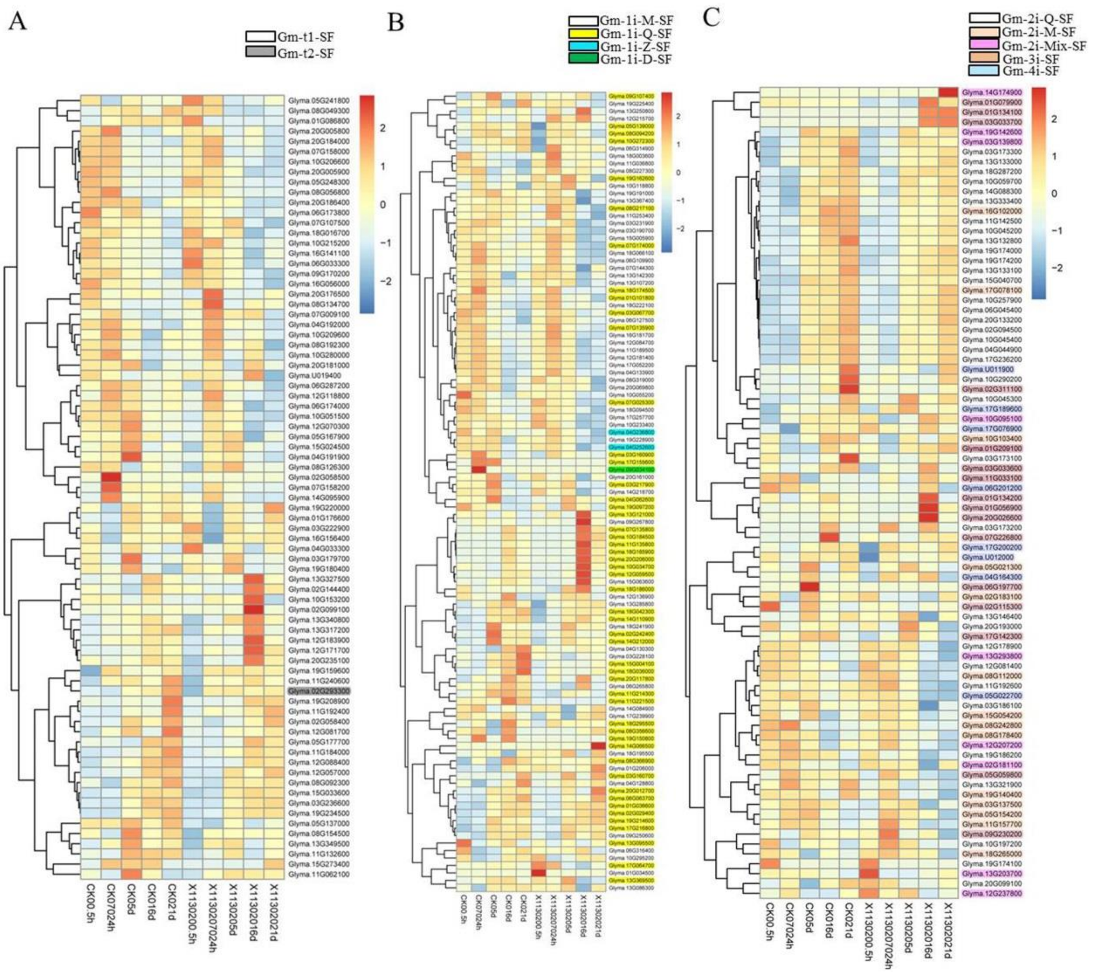


**Supplemental Figure S2: RNA-seq analysis of 321 soybean C2H2-ZFPs in soybean roots at different time points.** Heatmaps were produced based on our RNA-Seq results (Yuan et al., 2016a) using the pheatmap packages in R. (A) Heatmap for Gm-t1-SF and Gm-t2-SF genes. (B) Heatmap for Gm-1i-Q-SF, Gm-1i-M-SF, Gm-1i-Z-SF and Gm-1i-D-SF genes. (C) Heatmap for Gm-2i-Q-SF, Gm-2i-M-SF, Gm-2i-Mix-SF, Gm-3i-SF and Gm-4i-SF genes.
