# Supplementary material for: Genome-Wide Identification and Classification of Soybean C2H2 Zinc Finger Proteins and Their Expression Analysis in Legume-Rhizobium Symbiosis
Source: Front Microbiol. 2018 Feb 6;9:126. doi: 10.3389/fmicb.2018.00126 (PMC5807899; doi:10.3389/fmicb.2018.00126)
Supplement: Supplementary Figure S3 — The Expression analysis of M. truncatula C2H2-ZFPs in different root hairs and different nodules. [file Image3.pdf]

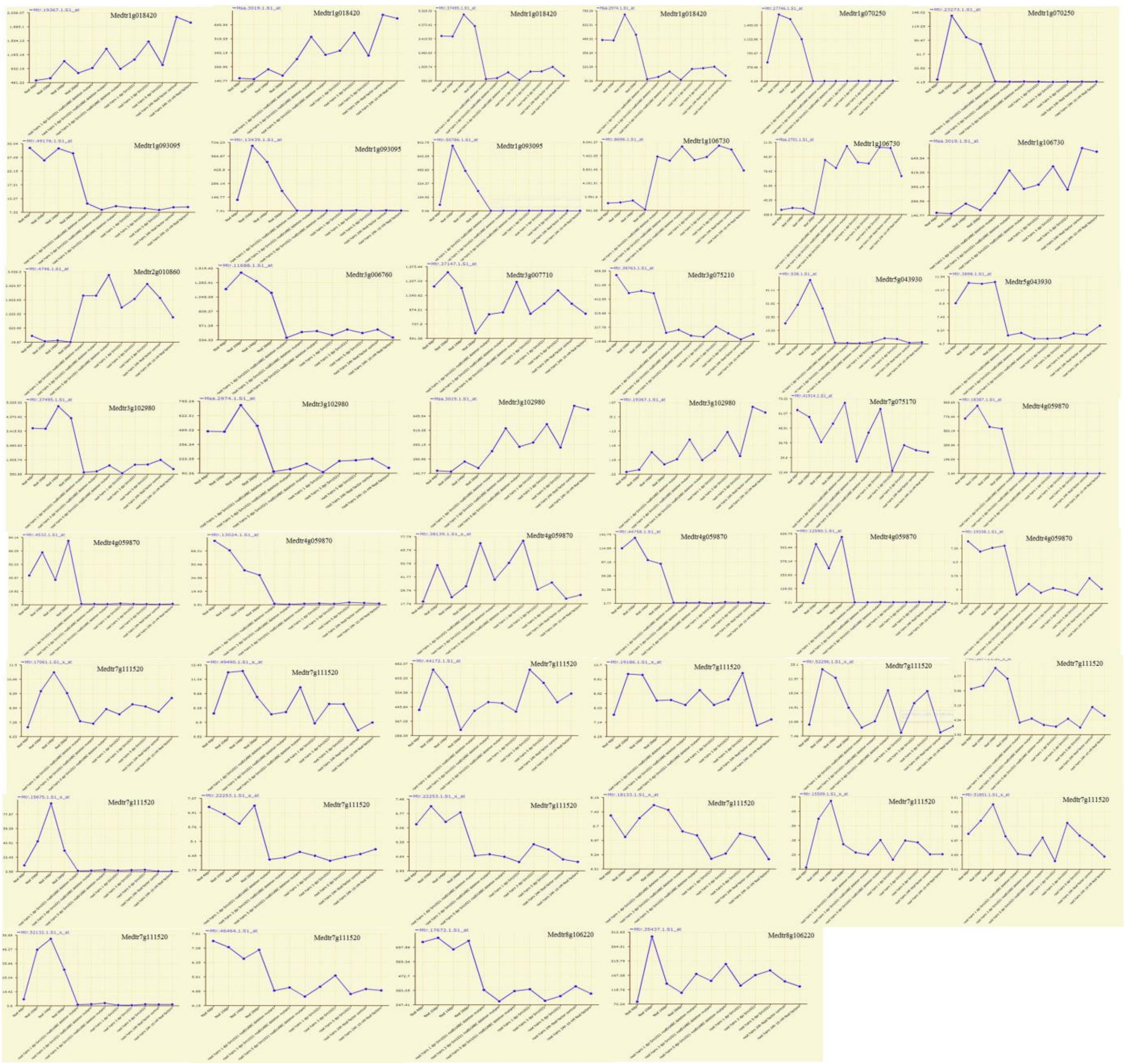

Supplemental Figure S3: The Expression analysis of *M. truncatula* C2H2-ZFPs in different root hairs and different nodules. The expression data were downloaded from *M. truncatula* Gene Expression Atlas database, and these genes contain 1~14 expression probeset IDs. Nod 4dpi, 4dN after rhizobial inoculation; Nod 10dpi, 10dN after rhizobial inoculation; Nod 14dpi, 14dN after rhizobial inoculation; Nod 28dpi, 28dN after rhizobial inoculation. The others are root hairs samples.
